# Gαi1 and Gαi3 mediate IL-11-induced signal transduction and are potential therapeutic targets for LUAD

**DOI:** 10.1038/s41419-026-08637-w

**Published:** 2026-04-25

**Authors:** Gaomeng Luo, Wenxuan Hu, Jian Yang, Zheng Lu, Yuan Cui, Weibiao Zeng, Hao Ding, Qifan Li, Zhike Chen, Xin Tong, Cheng Ding, Chun Xu, Jun Zhao

**Affiliations:** 1 Department of Thoracic Surgery, The First Affiliated Hospital of Soochow University, Suzhou Medical College of Soochow University, Suzhou, China; 2 Institute of Minimally Invasive Thoracic Cancer Therapy and Translational Research, Soochow University, Suzhou, China; 3 Department of Neurosurgery, Zhongnan Hospital of Wuhan University, Wuhan, China; 4 Department of Thyroid Vascular Surgery, The Eighth Affiliated Hospital of Southern Medical University, Guangdong Province, China

**Keywords:** Cell signalling, Tumour biomarkers, Non-small-cell lung cancer

## Abstract

Lung adenocarcinoma (LUAD) is the main histologic subtype of lung cancer, and its incidence is on the rise. However, since the vast majority of patients are already in advanced stages at the time of diagnosis, their 5-year survival rate is only 15%, so it is urgent to explore the mechanism of the development of LUAD and improve the survival time of patients. Interleukin-11 (IL-11), a member of the IL-6 cytokine family, has an influential role in the development and progression of a variety of tumors, but the specific molecular mechanisms that promote the malignant progression of LUAD are unknown. Here, we found that the IL-11-induced activation of Akt, Erk, and STAT3 could be inhibited by knocking out the expression of Gαi1/3. In contrast, overexpression of Gαi1/3 could enhance IL-11-induced signaling. The binding of Gαi1/3 to GP130 mediates IL-11-induced downstream activation of Akt-mTOR, Erk, and STAT3, which requires recruitment of Grb2-associated binding protein 1 (Gab1). In LUAD cells, shGαi1/3 inhibited cell growth, proliferation, and migration as well as blocked the tumor-promoting ability of IL-11. However, overexpression of Gαi1/3 enhanced the IL-11-induced cell growth, proliferation, and migration. ShGαi1/3 also inhibited the proliferation of LUAD cells in vivo. Overall, the findings of this study demonstrate the Gαi1 and Gαi3 are critical for IL-11 signal transduction. Moreover, we reveal that Gαi1 and Gαi3 are highly expressed and associated with poor overall survival in lung adenocarcinoma and may thus act as potential therapeutic targets in LUAD. These results provide a novel therapeutic strategy for LUAD patients with upregulated IL-11 expression.

## Introduction

Cancer development and progression, and the immune response against tumors are regulated by a number of different factors, including a variety of interleukin [1–3]. Interleukin 11 (IL-11), a member of the IL-6 cytokine family, shares the same protein fold as the other family members and consists of four alpha helices arranged in an upside-down topology [4]. IL-11 was initially found to be a soluble factor present in the supernatants of the PU34 cell line that stimulates proliferation of plasmacytoma cells when applied to them [4]. As a member of the IL-6 family of cytokines, IL-11 is best known for its role in promoting megakaryopoiesis, erythropoiesis, and thrombogenesis. Human IL-11 recombinant protein (rhIL-11) has been used clinically to treat chemotherapy-induced thrombocytopenia [5]. IL-11 plays an important role in fetal lung development, but serum levels of the protein are often undetectable in healthy adults [6]. However, IL-11 overexpression is often detected in disease states such as viral infections, fibrosis, and various cancers, suggesting that IL-11 is involved in a variety of disease developmental processes [7, 8]. In many cases, its expression correlates with disease severity.

Through its coupling with IL-11Rα and GP130 in a 2:2:2 stoichiometry, IL-11 activates the JAK-STAT3, RAS-RAF-ERK, and PI3K-AKT signaling pathways. This in turn drives tumorigenesis and cancer progression [8, 9]. Matadeen et al. observed structural and dynamic changes in the IL-11-IL-11Rα-GP130 extracellular complex by cryo-electron microscopy at 30 A resolution, and their analysis revealed a series of conformational changes that may play a role in signaling triggering [10]. Metcalfe et al. described an IL-11 variant, IL-11 mutant protein, based on the structure of the human IL-11 signaling complex, which effectively inhibits IL-11 signaling [11]. Significantly higher concentrations of IL-11 were detected in serum and exhaled breath condensate specimens in patients with non-small cell lung cancer (NSCLC). IL-11 expression is positively correlated with NSCLC lymph node metastasis, distant metastasis, tumor node metastasis stage, and degree of tumor differentiation [12]. IL-11 induces STAT3 phosphorylation and increases the expression of the anti-apoptotic proteins Bcl-2 and Survivin in lung Adenocarcinoma (LUAD) cells [13]. Thus, inhibition of IL-11 signaling could be a therapeutic strategy for LUAD.

Heterotrimeric G proteins consist of three subunits, α, β, and γ, of which the Gα subunit plays a key role in signaling. When G-protein coupled receptors (GPCRs) are activated, GDP dissociates from the Gα subunit and is replaced by GTP, resulting in the separation of Gα from the Gβγ dimer, which in turn activates downstream effectors. The G protein family is categorized according to its Gα subunit and divided into four families based on their sequence and functional similarity: Gαs, Gαi, Gαq, and Gα12/13 [14]. Gαi proteins are mainly categorized into Gαi1, Gαi2, and Gαi3, which play key roles in a variety of biological processes and are closely associated with a variety of diseases [15]. In recent years, a large amount of literature has shown that Gαi proteins can mediate the signaling of a variety of cytokines [16–18].

In the present study, we aimed to determine the role of Gαi proteins in IL-11 signaling. By knocking down and overexpressing Gαi1 and Gαi3 in MEFs, we verified that they play a key role in the IL-11-mediated activation of STAT3, Akt, and Erk1/2. Additionally, we found that Gαi1/3 mediates IL-11 signaling by coupling with GP130 and Gab1 in the LUAD cell lines. We confirmed the oncogenic behavior of Gαi in LUAD through functional experiments in cellular and animal models, bioinformatics analysis, and immunohistochemistry performed on tissue microarrays. In conclusion, the results of the present study demonstrate that Gαi1 and Gαi3 proteins are required for IL-11 signaling, and the expression of Gαi1 in patients with LUAD is negatively associated with poor prognosis. Gαi1 and Gαi3 may thus act as potential prognostic biomarkers and therapeutic targets in LUAD.

## Materials and methods

### Cell culture

The human LUAD cell lines, A549 and H1299, were purchased from the Cell Bank of the Chinese Academy of Sciences, authenticated by STR profiling, and free of mycoplasma contamination [19]. Two cell lines were routinely cultured in a constant humidity incubator at 37 °C, 5% CO_2_, using RPMI 1640 medium (Corning, USA) containing 10% fetal bovine serum (Gibco, USA) supplemented with penicillin and streptomycin to maintain a sterile environment. Wild-type (WT), Gαi1 and Gαi3 double-knockout (DKO), Gαi1, Gαi2 or Gαi3 single-knockout (SKO) mouse embryonic fibroblasts (MEFs), as well as Gab1 KO MEFs, were obtained from the gift of Prof. Cao, Institute of Neurology, Soochow University [20]. MEFs were cultured in DMEM (Corning, USA) containing 10% FBS. Cells were starved in 0.5% FBS medium overnight, followed by the addition of 30 min of steady heat PBS for signaling analysis.

### Antibodies

The antibodies utilized in this study were: Mouse anti-GAPDH (Proteintech, Cat#60004-1-Ig), Mouse anti-IL-11 (Santa Cruz, Cat#sc-133062), Mouse anti-β-actin (Proteintech, Cat#66009-1-Ig), Mouse anti-Gαi1 (Santa Cruz, Cat#sc-515658), Rabbit anti-Gαi1 (Abcam, Cat#ab220277), Mouse anti-Gαi2 (Santa Cruz, Cat#sc-13534), Mouse anti-Gαi3 (Santa Cruz, Cat#sc-365422), Rabbit anti-Gαi3 (Proteintech, Cat#11641-1-AP), Mouse anti-GP130 (Santa Cruz, Cat#sc-9994), Rabbit anti-phospho-Gab1 Antibody (Cell Signaling Technology, Cat#3233), Rabbit anti-phospho-STAT3 (Cell Signaling Technology, Cat#9145), Rabbit anti-phospho-Akt (Cell Signaling Technology, Cat#9271), Rabbit anti-phospho-S6k (Cell Signaling Technology, Cat#9234), Rabbit anti-phospho-S6 (Cell Signaling Technology, Cat#4856), Rabbit anti-phospho-Erk1/2 (Cell Signaling Technology, Cat#9101), Rabbit anti-Gab1 Antibody (Cell Signaling Technology, Cat#3232), Mouse anti-STAT3 (Cell Signaling Technology, Cat#9139), Mouse anti-Akt1/2/3 (Santa Cruz, Cat#sc-56878), Mouse anti-S6k (Santa Cruz, Cat#sc-8418), Mouse anti-S6 (Santa Cruz, Cat#sc-74459), Mouse anti-Erk1/2 (Santa Cruz, Cat#sc-514302), Rabbit anti-Ki67 Antibody (Merk, Cat#SAB5700770), Rabbit anti-Cleaved-Caspase-3 Antibody (Cell Signaling Technology, Cat#9664), Rabbit anti-Cleaved-Caspase-9 Antibody (Cell Signaling Technology, Cat#9505).

### Human tissues

The 17 pairs of newly resected LUAD tissues and adjacent normal lung tissues were used for immediate analysis, with the adjacent normal tissue located at least 5 cm from the tumor margin [21]. The inclusion criteria for the samples were patients with a clear pathological diagnosis of LUAD who had not undergone preoperative treatments such as radiotherapy, chemotherapy, immunotherapy, or targeted therapy. All tissue samples underwent rigorous pathological examination and confirmation to ensure accuracy. Included in our specimen repository after obtaining informed consent from the patient. For detailed clinical information on the patients included, please refer to Supplementary Table 1. The study followed the ethical principles of the Declaration of Helsinki and was approved by the Ethics Committee of the First Affiliated Hospital of Soochow University.

### Data collection

The RNA expression and clinicopathologic profiles of the Cancer Genome Atlas (TCGA) program and genotype-tissue expression (GTEx) portal were downloaded from the UCSC Xena database (https://xenabrowser.net/datapages/) [22]. This was followed by plotting *IL-11* expression histograms, Kaplan–Meier (KM) survival curves, and receiver operating characteristic (ROC) curves using an online plotting site (https://www.xiantaozi.com/). The single-cell RNA (scRNA) sequencing in the tumor microenvironment was analyzed by employing the Tumor Immune Single-cell Hub (TISCH) (http://tisch.comp-genomics.org/) [23, 24]. Here, expression levels of *IL-11* were examined in different cell types at single-cell levels in NSCLC.

### GO and KEGG pathway enrichment analyses

The biological processes, cellular components, and molecular functions associated with *IL-11* were characterized by (Gene Ontology) GO enrichment. The biological function of *IL-11* in LUAD was assessed by (Kyoto Encyclopedia of Genes and Genomes) KEGG enrichment analyses. Drawing of GO and KEGG bubbles using the Bioinformatics Analysis Tools website (https://www.bioinformatics.com.cn) [25].

### Gene set enrichment analysis

Gene Set Enrichment Analysis (GSEA) was performed using the software GSEA 4.1.0 (Broad Institute, Cambridge, USA). According to the median *IL-11* expression level, patients with LUAD from the TCGA database were stratified into high- and low-*IL-11* expression subgroups for GSEA enrichment analysis, and 1000 permutations were performed for each gene set. Hallmark enrichment pathways were classified according to *P* values and normalized enrichment scores (NESs), and the significance of enrichment was defined by a false discovery rate (FDR) < 0.25, as recommended by the GSEA software documentation.

### RNA isolation and qRT-PCR

Total RNA was extracted from cells and tissues using TRIzol reagent (Thermo Fisher Scientific, USA), followed by cDNA synthesis with HiScript Q RT SuperMix (Vazyme, China) according to the manufacturer’s protocols. The mRNA expression levels of *Gαi1*, *Gαi2*, and *Gαi3* were quantified using *GAPDH* as the endogenous control, with relative expression values determined through the quantitative comparative Ct (ΔΔCt) method. The following primers were used: *Gαi1* (Forward primer: 5′-TTAGGGCTATGGGGAGGTTGA-3′; Reverse primer: 5′-GGTACTCTCGGGATCTGTTGAAA-3′); *Gαi2* (Forward:5′-TACCGGGCGGTTGTCTACA-3′; Reverse primer: 5′-GGGTCGGCAAAGTCGATCTG-3′); *Gαi3* (Forward primer: 5′-GACGGCTAAAGATTGACTTTGGG-3′; Reverse primer: CCGTTTAATCACTCCTGCTAGTT-3′); *IL-11* (Forward primer: TGCACAGCTGAGGGACAA; Reverse primer: AGGTAGGACAGTAGGTCCGC); *GAPDH* (Forward primer: 5′-ACAACTTTGGTATCGTGGAAGG-3′; Reverse primer: 5′-GCCATCACGCCACAGTTTC-3′).

### RNA sequencing analysis

Total RNA was extracted using the TRIzol reagent according to the manufacturer’s protocol. Libraries were constructed using VAHTS Universal V10 RNA-seq Library Prep Kit (Premixed Version) according to the manufacturer’s instructions. The libraries were sequenced on an Illumina Novaseq X Plus platform, and 150 bp paired-end reads were generated. Raw reads of fastq format were firstly processed using fastp, and the low quality reads were removed to obtain the clean reads. The clean reads were mapped to the reference genome using HISAT2. FPKM of each gene was calculated, and the read counts of each gene were obtained by HTSeq-count. The shIL-11-1 and shIL-11-2 sequences were “CCTACTGTCCTACCTGCGGCA” and “TGCACAGCTGAGGGACAAATT”, respectively.

### Immunofluorescence and immunohistochemical assays

For immunofluorescence assays, cells were seeded on glass slides overnight, after which they were fixed with 4% PFA for 20 min. The cells were then permeabilized with 0.5% Triton X-100 and blocked with 5% goat serum for 30 min. After washing three times with 1× PBS, the cells were incubated with mouse anti-GP130 (Santa Cruz, USA), rabbit anti-Gαi1 (Abcam, USA) or rabbit anti-Gαi3 (Proteintech, USA) antibodies overnight at 4 °C; this was followed by incubation with anti-mouse secondary antibody (Abcam, USA) and anti-rabbit secondary antibody (Abcam, USA) for 2 h. Nuclei were counterstained with DAPI. Fresh clinical specimens underwent fixation, gradient sucrose dehydration, and cryosectioning (10 μm thickness) prior to immunofluorescence processing. The frozen tissue sections were incubated with mouse anti-IL-11 antibody (Santa Cruz, USA) and anti-mouse secondary antibody, and finally counterstained with DAPI and photographed. The immunofluorescence staining procedure for Ki67 (Merck, USA) was consistent with the above. The experimental methods for immunohistochemical (IHC) staining of tissue microarrays have been described in detail [21].

### Western blotting and co-immunoprecipitation (Co-IP) assay

The methods used for western blotting and Co-IP and for quantifying the data obtained through these analyses have been extensively described previously [26]. Briefly, cells were first plated in complete medium. After 24 h of incubation, the cells were grown in 0.5% serum (starvation) overnight. After starvation, the cells were stimulated with medium containing the treatment (IL-11 50 ng/mL) of interest for different periods of time. Cell lysates were then collected for western blotting experiments, and samples were run on parallel (“sister”) gels or reincubated with other antibodies after stripping the antibodies using stripping buffer (Cwbio, China) to detect different proteins, as needed. Quantitative analysis of data obtained was performed using the ImageJ software. Uncropped immunoblot data have been provided in the Supplementary material 1.

For Co-IP, cell lysates were incubated with antibodies against GP130 or IgG on a rotator at 4 °C overnight. Protein A/G agarose beads (Santa Cruz, USA) were added and incubated at room temperature for 2 h to capture the immune complexes. Finally, immunoprecipitation containing target and interacting proteins was isolated from agarose beads for western blotting analysis.

### Molecule docking analysis

First, we downloaded the protein sequences for GP130, Gαi1, and Gαi3 from the UniProt website (https://www.uniprot.org/) [27]. Input protein sequences into AlphaFold3 website (https://alphafoldserver.com/welcome) for protein-protein interaction model simulation, and the model with the highest score was selected [28]. Visualization was performed using PyMOL software (https://pymol.org/).

### EdU analysis

The A549 and H1299 cells were inoculated in 24-well plates at a density of 5×10^4^ cells/well and received the appropriate treatments after a specific incubation length. The cell proliferation capacity was quantitatively assessed using the EdU Apollo594 kit (Ribo Bio, China). Quantitative analysis was performed using ImageJ software, and the proliferation level was characterized by calculating the number of EdU-positive cells as a percentage of the total number of DAPI-positive cells (EdU/DAPI ratio). Five randomly selected fields of view for each experimental group were statistically analyzed, and the results were expressed as mean ± standard deviation.

### Transwell migration and invasion assays

A total of 5 × 10^4^ cells were suspended in serum-free medium or treatment-specific solutions (1% FBS) and seeded into the upper compartment of 8-µm pore polycarbonate membrane inserts (Corning, USA). For migration assays, the lower chamber was filled with 600 µl of 20% FBS-containing medium as a chemoattractant. Invasion assays required prior coating of the membrane with Matrigel (Corning, USA) to simulate extracellular matrix penetration. Following an appropriate incubation period, non-migratory cells on the upper membrane surface were removed using cotton swabs. Transmigrated cells were fixed with 4% paraformaldehyde (PFA) for 20 min and stained with 1% crystal violet solution for 5 min. A minimum of five randomly selected microscopic fields per condition were imaged, with cell counts automated via ImageJ software.

### Mouse xenograft studies

BALB/c nude mice aged 5–6 weeks were purchased from the Experimental Animal Center of Soochow University, maintained and treated under specific pathogen-free conditions. Mice were randomly divided into two groups (*n* = 6). For tumor inoculation, a single-cell suspension containing 5 × 10^6 A549 cells in 200 µl PBS was subcutaneously injected into the mid-axillary region using a needle. Subcutaneously xenografted tumor growth was longitudinally monitored through caliper-based volumetric measurements at 5 day intervals, with surgical excision performed on day 30 post-implantation. The cohorts of mice were sacrificed when the biggest tumors of the respective controls were ~1000 mm^3^. The animal experiment was approved by the Institutional Animal Care and Use Committee (IACUC) and ethics review committee of Soochow University.

### In vivo metastasis assays

BALB/c nude mice aged 5–6 weeks were purchased from the Experimental Animal Center of Soochow University, maintained, and treated under specific pathogen-free conditions. Following the randomization principle, each group comprised 5 mice. Mice received tail vein injections of A549 cells suspended in 200 μl PBS (3×10^6 cells/mouse). Nude mice were monitored periodically and euthanized 8 weeks post-injection. Lungs were excised and fixed in Bouin’s solution for metastatic nodule analysis. The animal experiment was approved by the Institutional Animal Care and Use Committee (IACUC) and ethics review committee of Soochow University.

### Statistical analyses

All in vitro experiments were repeated at least three times, and data are presented as mean ± SD. Unpaired t test (two-tailed) was used to compare data between the two groups. One-way or two-way ANOVA was chosen for multiple comparisons where appropriate. All post hoc analysis were performed using either Tukey’s or Šídák’s multiple comparison test, where appropriate. *P* values < 0.05 were considered to be statistically significant. The GraphPad Prism 10 software was used for statistical analysis and statistical chart drawing.

## Results

### *IL-11* was overexpressed and positively associated with poor prognosis in LUAD

**Fig. 1:**
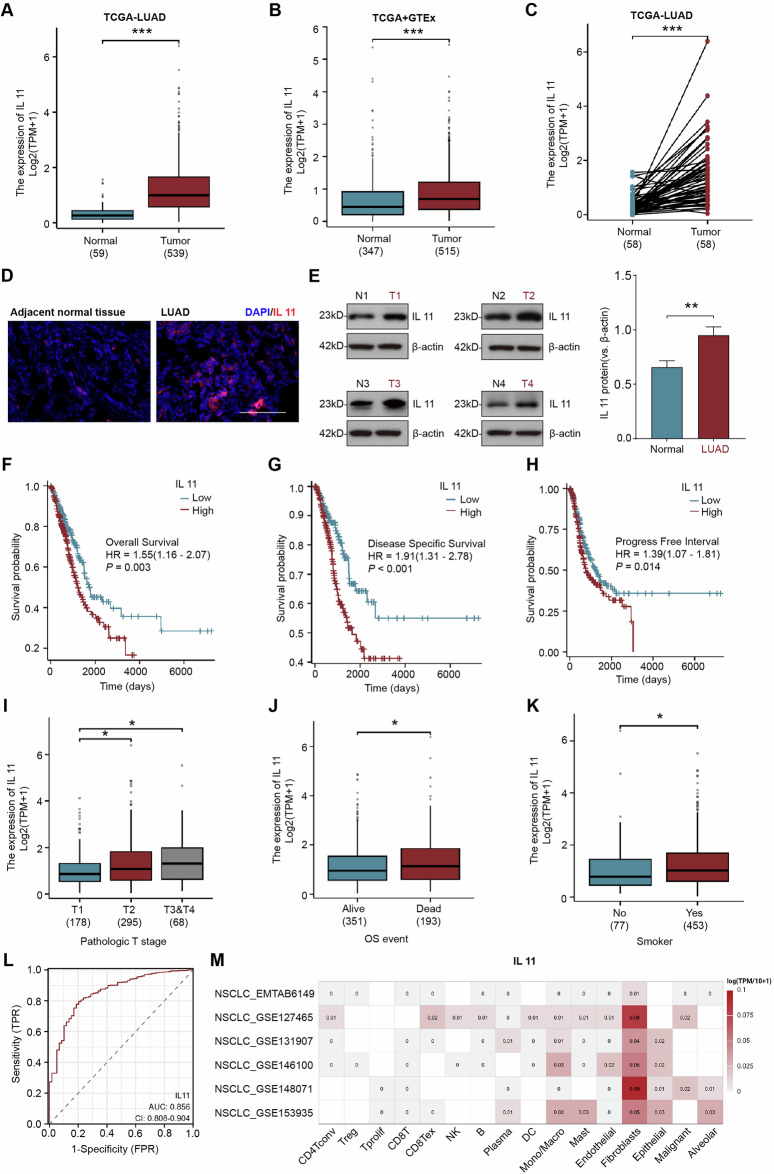
*IL-11* overexpression in human LUAD correlates with key clinical parameters of the patients. The cancer genome atlas lung adenocarcinoma (TCGA-LUAD) cohort showing *IL-11* expression levels in LUAD and normal lung tissues (**A**). Integration of TCGA database with genotype-tissue expression (GTEx) project for *IL-11* expression levels in LUAD tissues and normal tissues (**B**). Statistical analyses of *IL-11* expression levels in 58 pairs of LUAD tissues and paired adjacent normal tissues from the TCGA database (**C**). Immunofluorescence staining of IL-11 in human LUAD and adjacent normal tissues derived from clinical specimens (**D**). Proteins were extracted from LUAD patient tumor tissues (“T”) and adjacent normal tissues (“N”), and IL-11 protein levels were detected by Western blot analysis (**E**). The Kaplan-Meier survival analyses demonstrated the associations between *IL-11* expression and overall survival (**F**), disease specific survival (**G**), and progress free interval (**H**) of LUAD patients. Subgroup analyses of *IL-11* mRNA expression levels in patients with LUAD in terms of the listed clinical characteristics (**I**–**K**). The receiver operating characteristic (ROC) curves assessing *IL-11* expression for its predictive value in LUAD patients (**L**). Heatmap of *IL-11* expression in different cell types using TISCH database (**M**). *P < 0.05; **P < 0.01; ***P < 0.001. Scale bar = 100 µm.

First, we compared the gene expression levels of *IL-11* between 539 LUAD tumor tissues and 59 normal lung tissues from TCGA data and found that *IL-11* was highly expressed in LUAD (Fig. 1A). After integrating *IL-11* expression data from normal lung tissues in the GTEx database, IL-11 was still upregulated in LUAD tissues (Fig. 1B). Similarly, by comparing the expression levels of *IL-11* in 58 pairs of LUAD and normal lung tissues, we found that the difference in *IL-11* expression between the two tissue types was highly significant (Fig. 1C). To confirm the results of the analysis, we performed immunofluorescence analysis of clinical LUAD specimens; this analysis also revealed a higher expression of IL-11 in LUAD compared to that in normal lung tissue (Fig. 1D). Western blot results also demonstrated that IL-11 protein elevation was also detected in LUAD tissues, whereas its expression was relatively low in cancer-surrounding normal tissues (Fig. 1E). Kaplan–Meier survival curves indicated that elevated *IL-11* levels in LUAD are associated with poorer overall survival, disease specific survival and progress free interval (Fig. 1F–H). In LUAD tissues, the expression of *IL-11* was elevated with pathological T stage (Fig. 1I); however, it was not related to N stage and M stage (SFig. 1A, B). *IL-11* expression was also significantly higher in tissues from dead LUAD patients (“Dead”) than in LUAD tissues from surviving patients (“Alive”) (Fig. 1J). In addition, *IL-11* was overexpressed in LUAD tissues from smoking patients compared with non-smoking patients (Fig. 1K). There was no difference in *IL-11* expression between male and female LUAD patients (SFig. 1C). The ROC curve analysis indicated that *IL-11* had excellent diagnostic efficiency in differentiating between LUAD and normal tissues. The area under the curve (AUC) was 0.856 for LUAD patients (Fig. 1L), suggesting its potential as a diagnostic biomarker. The potential role of *IL-11* in tumor microenvironment was further explored through estimating its expression at the single cell levels in various cell types based on the TISCH (Tumor Immune Single-cell Hub) database. The results in Fig. 1M showed that *IL-11* was highly expressed in fibroblasts and epithelial cells among the six GSE databases, indicating that IL-11 was mainly derived from these two cell types in lung cancer tissues. These findings collectively establish that *IL-11* is consistently overexpressed in LUAD and is strongly correlated with poor overall survival.

### The functional significance of *IL-11* in LUAD

**Fig. 2:**
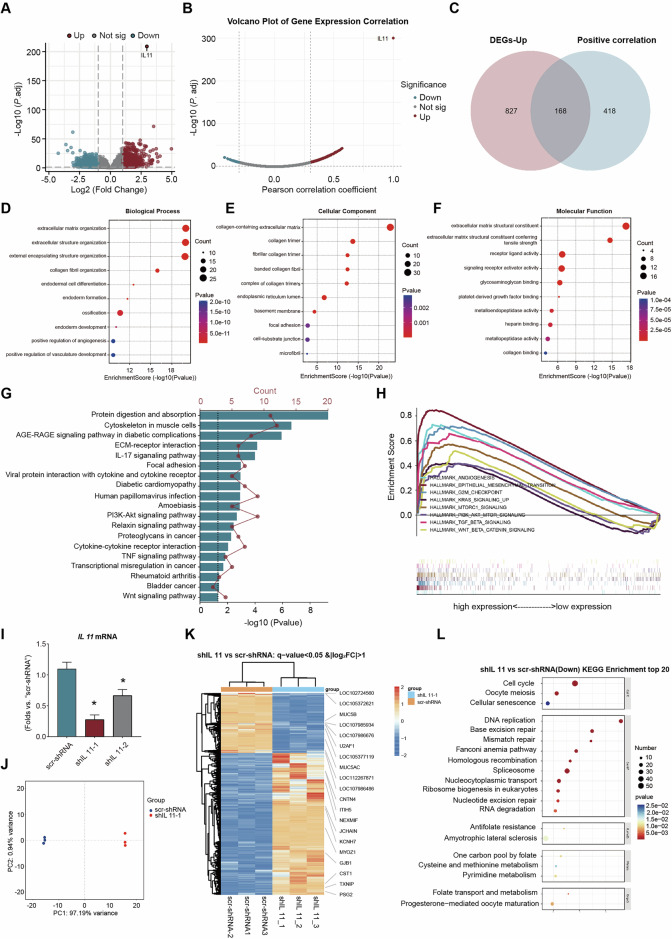
The functional significance of IL-11 in LUAD. The differentially expressed genes (DEGs) between the high-expression and low-expression groups of *IL-11* in TCGA-LUAD cohort were presented (**A**). *IL-11* co-expressed genes in LUAD from TCGA database were shown (**B**). The venn diagram illustrated the overlapping genes of *IL-11* positive correlation expressed genes and up DEGs from TCGA-LUAD cohort (**C**). Biological process (**D**), cellular component (**E**), molecular function (**F**) and Kyoto Encyclopedia of Genes and Genomes (KEGG) (**G**) enrichment pathways of these overlapping genes were presented. The results of GSEA enrichment analysis of *IL-11*-related differential genes (**H**). Quantitative reverse transcription polymerase chain reaction (qRT-PCR) analyses *IL-11* expression levels in *IL-11*-silenced or control A549 cells, which were transfected with short hairpin RNA (shIL-11-1 or shIL-11-2) or a scrambled sequence (scr-shRNA), GAPDH was used as an internal control (**I**). Principal component analysis plot of sequencing results (**J**). Heat map of gene expression, in which red indicates relatively high expression genes and blue indicates relatively low expression genes (**K**). KEGG enrichment analysis showed the top 20 KEGG enrichment pathways (**L**).

An extensive evaluation of *IL-11*’s functional significance in LUAD was conducted using data from TCGA. Firstly, differential expression analysis was performed, segregating LUAD samples into high and low *IL-11* expression groups based on the median *IL-11* expression level, revealing 2755 differentially expressed genes (DEGs) meeting log_2_(Fold Change) threshold surpassing 1 or below −1, with adjusted P-value below 0.05 (Fig. 2A and Supplementary Table 2). After that, Pearson correlation analysis identified 645 genes in LUAD that were significantly associated with IL-11, consistent with the criterion of correlation coefficients above 0.3 or below −0.3 with adjusted *p*-value of less than 0.05 (Fig. 2B and Supplementary Table 3). We obtained 168 genes by taking the intersection of 995 genes up-regulated in the high *IL-11* expression group with 586 genes whose expression was positively correlated with *IL-11* expression (Fig. 2C). We subsequently analyzed 168 genes for GO (Fig. 2D–F) and KEGG (Fig. 2G) enrichment. Functional annotation through GO/KEGG enrichment profiling identified significant enrichment in positive regulation of angiogenesis, signaling receptor activator activity, PI3K-Akt signaling pathway, proteoglycans in cancer, cytokine-cytokine receptor interaction, and transcriptional misregulation in cancer. To further explore the molecular mechanism underlying the role of *IL-11* in LUAD, we analyzed connections between *IL-11* and hallmark pathways by GSEA. The results showed that multiple hallmark pathways were associated with *IL-11* expression, including those related to angiogenesis, epithelial mesenchymal transition, G2M checkpoint, KRAS signaling up, mTORC1 signaling, PI3K/Akt/mTOR signaling, TGF-β signaling, and WNT/β-catenin signaling (Fig. 2H and SFig. 2A–H).

We subsequently downregulated *IL-11* expression in the A549 cell line. Knockdown effects were validated by qRT-PCR (Fig. 2I). The A549 shIL-11-1 cell line, which exhibited the most effective *IL-11* knockdown, was selected for high-throughput transcriptomic analysis. Principal component analysis demonstrated excellent differentiation between the A549 scr-shRNA and shIL-11 groups (Fig. 2J). Based on q < 0.05 and |log_2_ FC | > 1, compared to the control group, 3302 genes were upregulated and 1394 genes were downregulated following *IL-11* knockdown (Fig. 2K and Supplementary Table 4). Next, we performed KEGG enrichment analysis on the 1,394 downregulated genes (Fig. 2L). The results indicated that these downregulated genes were associated with pathways such as the cell cycle, DNA replication, nuclear-cytoplasmic transport, and cellular senescence. These results further suggest that IL-11 plays a tumor-promoting role in LUAD and that inhibition of IL-11-induced signaling may help to inhibit the progression of LUAD.

### Gαi1 and Gαi3 double knockout inhibited the IL-11-induced Akt, Erk, and STAT3 activation in (mouse embryonic fibroblasts) MEFs

**Fig. 3:**
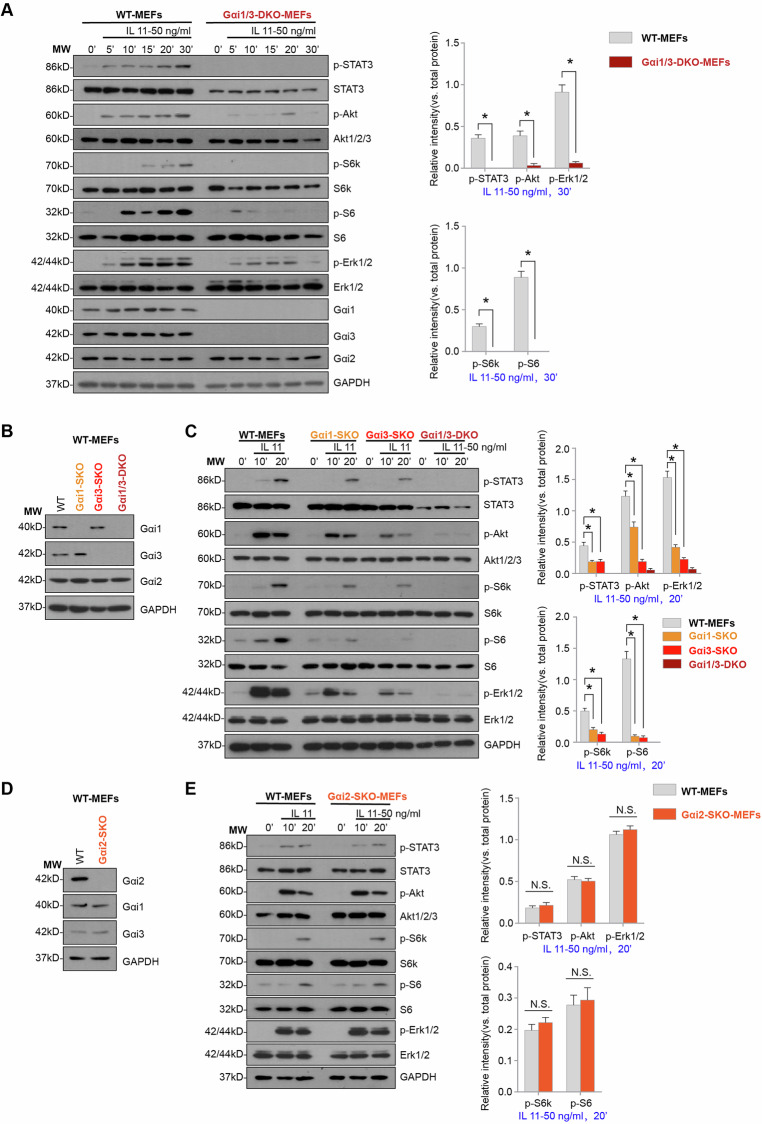
Gαi1 and Gαi3 double knockout inhibited IL-11-induced Akt, Erk, and STAT3 activation in MEFs. Wild type (WT), Gαi1 and Gαi3 double knock out (DKO) (**A**), Gαi1 or Gαi3 single knockout (SKO) (**C**), and Gαi2 SKO (**E**) MEFs were treated with IL-11 (at the indicated concentrations for the indicated time durations), and their total cell lysates were assessed for the expression of the listed proteins using western blotting. **B** shows the expression levels of the listed proteins in Gαi1 SKO and Gαi3 SKO MEFs. **C** was analyzed using a one-way ANOVA (Comparing p-STAT3 levels across multiple groups, *n* = 5 per group, one-way ANOVA, *F*_3, 16_ = 131.7, *P* < 0.0001, Tukey’s test, WT vs Gαi1 SKO, *P* < 0.0001, WT vs Gαi3 SKO, *P* < 0.0001; Comparing p-Akt levels across multiple groups, *n* = 5 per group, one-way ANOVA, *F*_3, 16_ = 370.9, *P* < 0.0001, Tukey’s test, WT vs Gαi1 SKO, *P* < 0.0001, WT vs Gαi3 SKO, *P* < 0.0001; Comparing p-Erk1/2 levels across multiple groups, *n* = 5 per group, welch’s ANOVA, *W*(DFn, DFd) = 346.0(3.0, 8.435), *P* < 0.0001, Dunnett’s T3 test, WT vs Gαi1 SKO, *P* < 0.0001, WT vs Gαi3 SKO, *P* < 0.0001; Comparing p-S6k levels across multiple groups, *n* = 5 per group, one-way ANOVA, *F*_3, 16_ = 226.0, *P* < 0.0001, Tukey’s test, WT vs Gαi1 SKO, *P* < 0.0001, WT vs Gαi3 SKO, *P* < 0.0001; Comparing p-S6 levels across multiple groups, *n* = 5 per group, one-way ANOVA, *F*_3, 16_ = 545.4, *P* < 0.0001, Tukey’s test, WT vs Gαi1 SKO, *P* < 0.0001, WT vs Gαi3 SKO, *P* < 0.0001). **D** shows the expression levels of the listed proteins in Gαi2 single knockout MEFs. “MW” stands for molecular weight (Same for all Figures). *P < 0.05. “N.S.” indicates *P* > 0.05.

Gαi proteins can mediate the signaling of a variety of cytokines [17, 18, 29]. To delineate the functional requirement of Gαi isoforms in IL-11 signaling, wild-type (WT) and Gαi1 and Gαi3 double knockout (Gαi1/3 DKO) MEFs were treated with IL-11 (50 ng/ml) for 5–30 min. Next, we used western blotting to assess the activation levels of IL-11 downstream signaling proteins and found that IL-11 significantly activated the phosphorylation levels of STAT3, Akt (Ser473), and Erk1/2 in MEFs. Moreover, we found that the activation of these phosphorylation levels was significantly impaired after Gαi1/3 knockdown (Fig. 3A). In addition, the phosphorylation levels of S6K and S6 downstream of AKT were inhibited in Gαi1/3 DKO MEFs.

Next, we constructed Gαi1, Gαi2, and Gαi3 SKO (single knockout) MEFs and treated them with IL-11 (50 ng/ml) for 10 and 20 min. We found that Gαi1 SKO and Gαi3 SKO in MEFs slightly inhibited IL-11-activated STAT3, Akt (Ser473), and Erk1/2, while Gαi1/3 DKO caused a more obvious inhibition of these downstream proteins of IL-6, almost completely blocking their phosphorylation (Fig. 3B, C). However, Gαi2 SKO in MEFs did not inhibit IL-11 signaling (Fig. 3D, E). These results suggest that IL-11-induced signaling activation is inhibited by Gαi1 and Gαi3 knockout but not Gαi2 knockout.

### Gαi1 and Gαi3 were required for IL-11-induced Akt, Erk, and STAT3 activation in MEFs

**Fig. 4:**
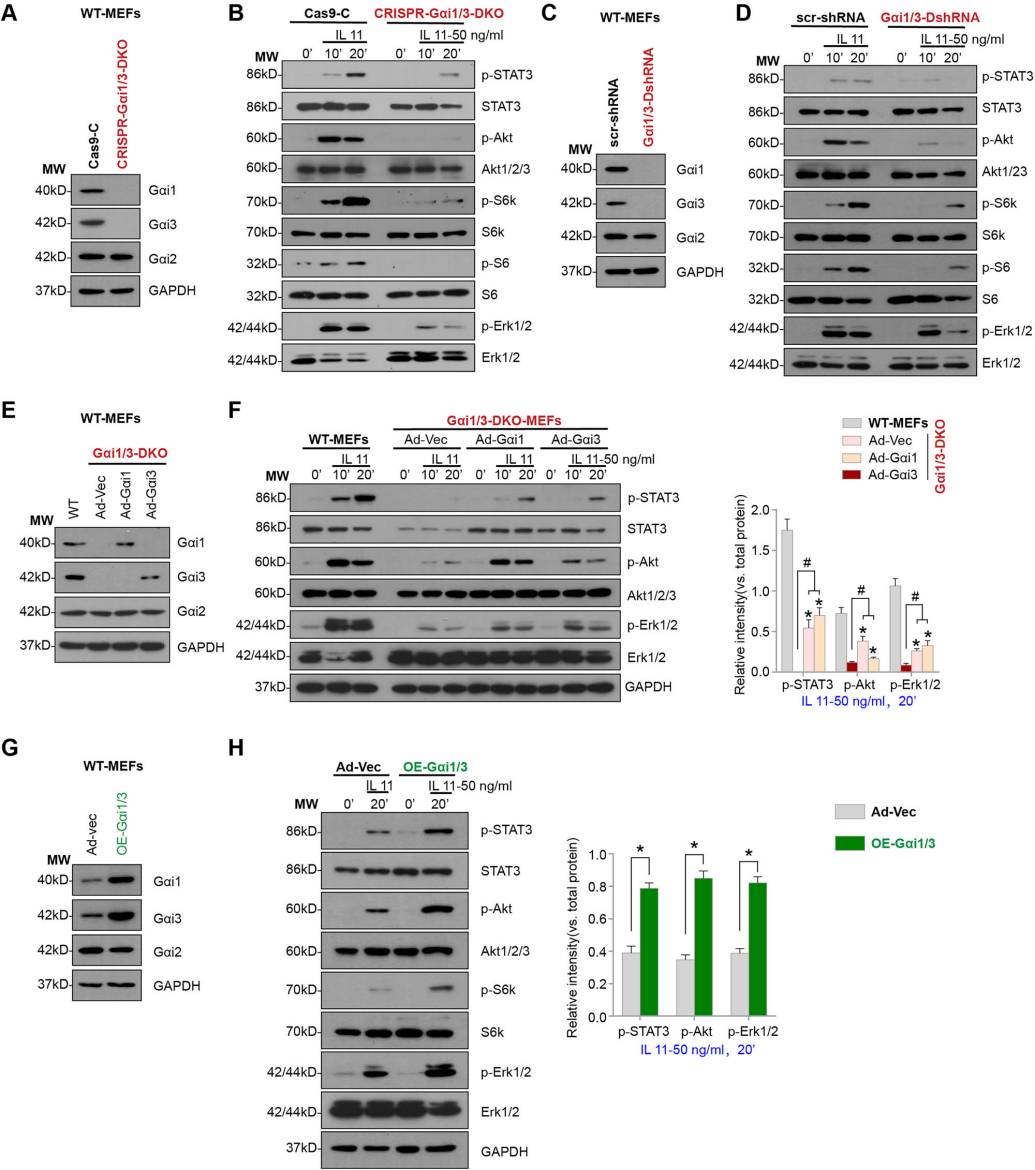
Gαi1 and Gαi3 were required for IL-11-induced Akt, Erk, and STAT3 activation in MEFs. WT-MEFs with Gαi1 and Gαi3 CRISPR/Cas9 KO constructs (“CRISPR/Cas9-Gαi1/3-DKO”) (**A**, **B**), WT-MEFs containing scramble control shRNA (“scr-shRNA”) or lentiviral Gαi1 or Gαi3 shRNA (“Gαi1/3 DshRNA”) (**C**, **D**) were treated with IL-11 (50 ng/mL) for the indicated time periods, and the expression levels of the listed proteins in their total cell lysates were assessed using western blotting. DKO-MEFs (**E**, **F**) or WT-MEFs (**G**, **H**) were transiently transfected with the adenoviral Gαi1 construct (“Ad-Gαi1”), the adenoviral Gαi3 construct (“Ad-Gαi3”), or the empty vector (“Ad-Vec”) and treated with IL-11 (50 ng/mL) for the indicated time periods; the expression levels of the listed proteins in these cells were then assessed using western blotting. Figure **F** was analyzed using a one-way ANOVA (Comparing p-STAT3 levels across multiple groups, *n* = 5 per group, one-way ANOVA, *F*_3, 16_ = 275.1, *P* < 0.0001, Tukey’s test, DKO vs AD-Gαi1, *P* < 0.0001, DKO vs AD-Gαi3, *P* < 0.0001; Comparing p-Akt levels across multiple groups, *n* = 5 per group, one-way ANOVA, *F*_3, 16_ = 160.1, *P* < 0.0001, Tukey’s test, DKO vs AD-Gαi1, *P* < 0.0001, DKO vs AD-Gαi3, *P* < 0.0001; Comparing p-Erk1/2 levels across multiple groups, *n* = 5 per group, one-way ANOVA, *F*_3, 16_ = 284.3, *P* < 0.0001, Tukey’s test, DKO vs AD-Gαi1, *P* < 0.0001, DKO vs AD-Gαi3, *P* < 0.0001). *P < 0.05; #*P* < 0.05.

To verify the importance of Gαi1 and Gαi3 proteins for IL-11 signaling, we used CRISPR/Cas9 gene editing to knock out Gαi1 and Gαi3 proteins in MEFs. Figure 4A confirms the stable knockdown of Gαi1 and Gαi3 in CRISPR/Cas9-Gαi1/3 DKO MEFs. The IL-11-induced phosphorylation of STAT3, Akt (Ser473), and Erk1/2 was found to be abolished in CRISPR/Cas9-Gαi1/3 DKO MEFs (Fig. 4B). Next, we interfered with the expression of Gαi1 and Gαi3 in MEFs using shRNAs (Fig. 4C). The IL-11-induced phosphorylation levels of STAT3, Akt, and Erk1/2 were found to be significantly inhibited in Gαi1/3 DshRNA-treated MEFs (Fig. 4D).

Moreover, we introduced adenoviral Gαi1 expression constructs (“Ad-Gαi1”) or adenoviral Gαi3 expression constructs (“Ad-Gαi3”) into the Gαi1/3 DKO MEFs to test whether ectopic expression of Gαi1 or Gαi3 could rescue IL-11 signaling, respectively (Fig. 4E). Western blotting demonstrated that ectopic expression of Gαi1 or Gαi3 could partially rescue IL-11 signaling (Fig. 4F). We also constructed MEFs with stable overexpression of Gαi1 or Gαi3 (“OE-Gαi1/3”). Gαi1/3 protein expression was significantly increased in OE-Gαi1/3 MEFs (Fig. 4G). The IL-11-induced phosphorylation levels of STAT3, Akt, and Erk1/2 were significantly increased in Gαi1/3 overexpressing MEFs (Fig. 4H). These findings suggest that Gαi1 and Gαi3 protein overexpression in MEFs can enhance IL-11-induced signaling.

### Gαi1/3 protein mediated IL-11 signaling by forming a complex with GP130 and Gab1

**Fig. 5:**
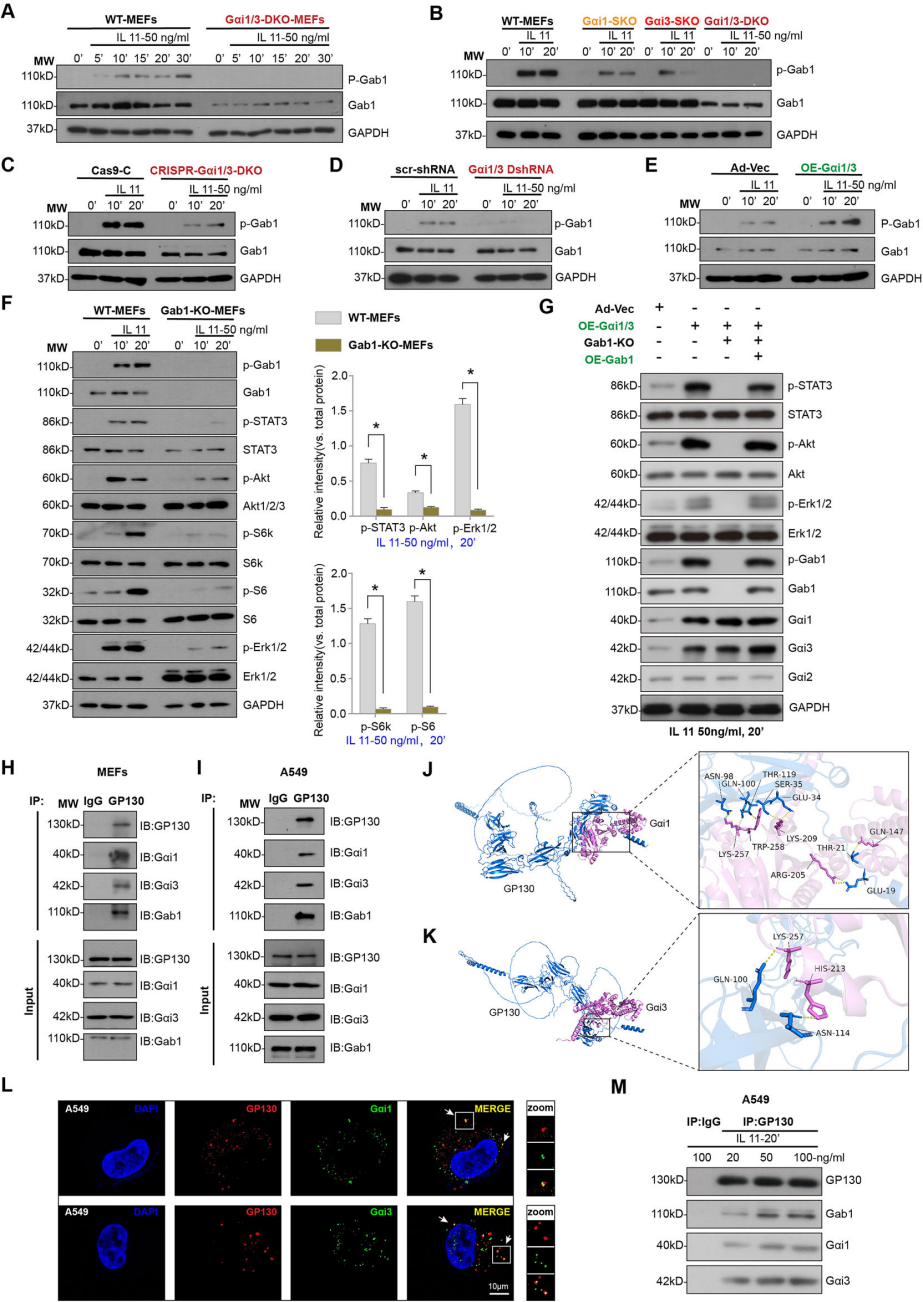
Gαi1/3 mediated IL-11-induced signaling by forming a complex with GP130 and Gab1. The phosphorylation levels of Gab1 after IL-11 treatment were detected in WT, Gαi1/3-DKO (**A**), Gαi1-SKO, Gαi3-SKO (**B**), CRISPR/Cas9-Gαi1/3-DKO (**C**), Gαi1/3-DshRNA (**D**), and OE-Gαi1/3 (**E**) MEFs. WT and Gab1-KO MEFs were treated with IL-11 (50 ng/mL) for the indicated time periods, and the expression levels of the indicated proteins in total cell lysates were assessed using western blotting (**F**). Treatment of MEFs with 50 ng/ml IL-11 for 20 min followed by western blotting to detect the expression of relevant proteins (**G**). CO-IP was performed in MEFs (**H**) and A549 (**I**) cells to examine the binding between GP130, Gαi1, Gαi3, and Gab1. An anti-immunoglobulin G (IgG) antibody was used as a negative control. AlphaFold3 analysis predicted the interaction between GP130 and Gαi1 (**J**). AlphaFold3 analysis predicted the interaction between GP130 and Gαi3 (**K**). A549 cells were stained with GP130, Gαi1, and Gαi3 antibodies. Cell nuclei were stained and visualized with DAPI (blue) (**L**). A549 cells were treated with IL-11 (20, 50, or 100 ng/mL) and cultivated for 20 min, GP130 association with Gab1 and Gαi1/3 was examined by Co-IP assays (**M**). Zoomed images of framed regions. *P < 0.05. Scale bar = 10 µm.

Our previous studies revealed that Gαi1/3 protein can mediate the signaling of a variety of cytokines through Grb2-associated binder 1 (Gab1) [30, 31]. Therefore, we assessed whether Gab1 is required for IL-11 signaling. First, we found that IL-11 could mediate Gab1 phosphorylation, but this phosphorylation was significantly inhibited in Gαi1/3 DKO MEFs. This suggested that Gab1 was downstream of Gαi1/3 and participated in the IL-11 signaling pathway (Fig. 5A). Furthermore, the IL-11-induced phosphorylation of Gab1 was found to be inhibited in Gαi1 SKO, Gαi1 SKO, CRISPR/Cas9-Gαi1/3 DKO, and Gαi1/3-DshRNA MEFs (Fig. 5B–D). Moreover, Gab1 phosphorylation was more strongly blocked in Gαi1/3 DKO MEFs than in Gαi1 SKO and Gαi3 SKO MEFs (Fig. 5B). Overexpression of Gαi1/3 enhanced the IL-11-mediated phosphorylation of Gab1 (Fig. 5E). Upon Gab1 knockout in MEFs, we found that the IL-11-induced activation of the Jak2/STAT3, PI3K/Akt, and Erk/MAPK signaling pathways was significantly inhibited (Fig. 5F). In rescue experiments, overexpression of Gαi1/3 promoted IL-11-induced signal transduction only in the presence of Gab1 protein (Fig. 5G). These results suggested that Gab1 is located downstream of Gαi1 and Gαi3 proteins in IL-11-induced signaling.

Since Gαi and Gab1 can be recruited by RTK receptors to mediate the activation of downstream PI3K-AKT-mTORC1 signaling, we performed Co-IP experiments to verify whether Gαi and Gab1 can bind to GP130, the receptor of IL-11. Endogenous GP130 was found to co-precipitate with Gαi1, Gαi3, and Gab1 in MEFs (Fig. 5H). This co-precipitation was also demonstrated in the A549 cell line (Fig. 5I). Additionally, AlphaFold3 predicted that GP130 could interact with Gαi1 (Fig. 5J) and Gαi3 (Fig. 5K). Confocal imaging also showed co-localisation of GP130 with Gαi1 and Gαi3 in A549 cells, supporting the interaction between GP130 and Gαi proteins in LUAD cells (Fig. 5L). Co-IP assay results in A549 cells revealed that IL-11 promoted IL-11-GP130-Gαi1/3-Gab1 association (Fig. 5M). Overall, these findings indicate that Gαi1/3 coupled with Gp130 mediated the IL-11-induced activation of downstream STAT3, Akt, and Erk1/2 via Gab1.

### Interference with Gαi1 and Gαi3 expression could inhibit the proliferation, migration, and invasion of LUAD cells in vitro and block the cancer-promoting ability of IL-11

**Fig. 6:**
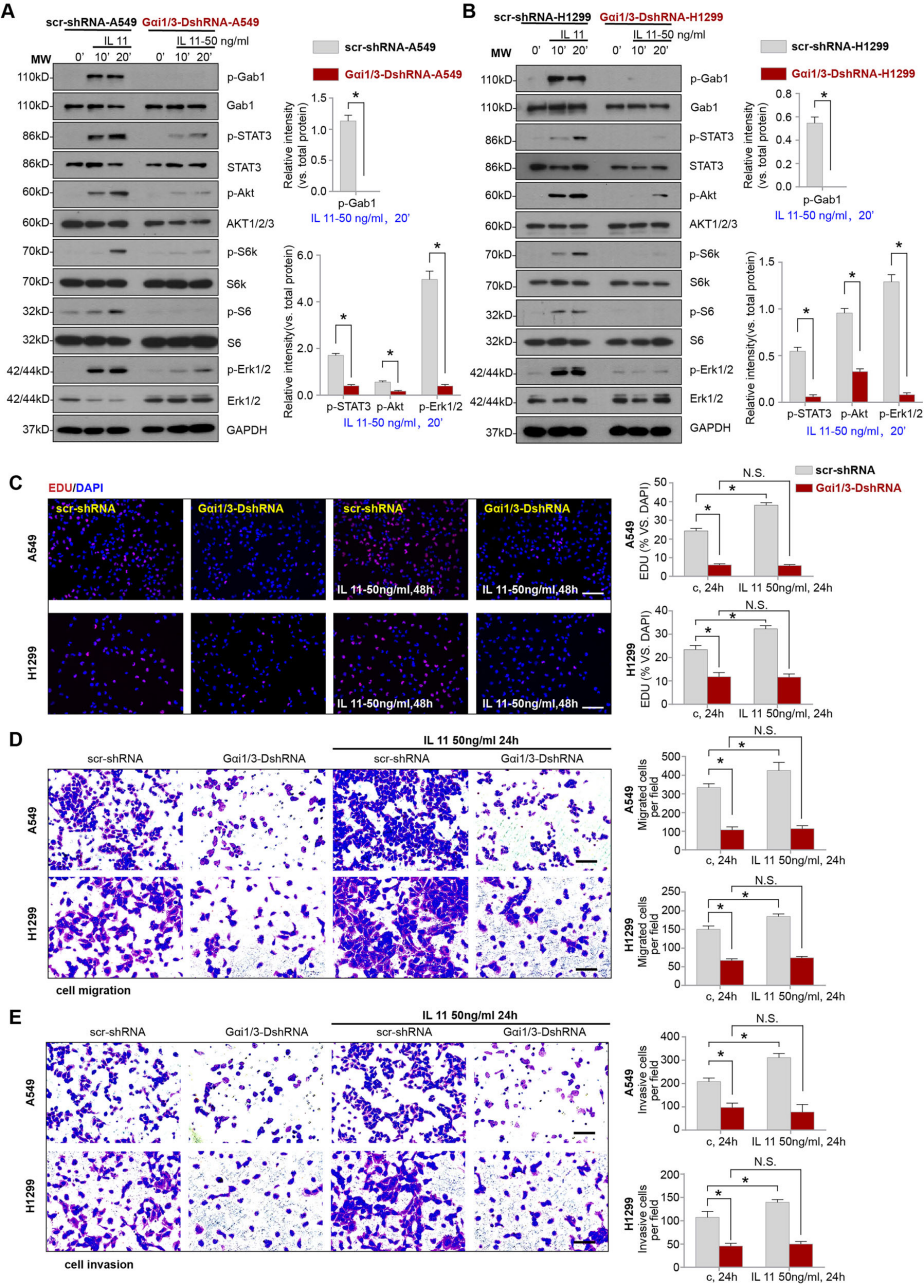
Interference with Gαi1 and Gαi3 could inhibit the proliferation, migration, and invasion abilities of LUAD cells in vitro and block the cancer-promoting ability of IL-11. A549 (**A**) or H1299 (**B**) cells containing scr-shRNA and Gαi1/3-DshRNA were treated with 50 ng/ml IL-11 at 0 min, 10 min, and 20 min, and the expression levels of the listed proteins were assessed using western blotting. After treatment with or without 50 ng/ml IL-11 for 48 h, the proliferation abilities of the abovementioned cell groups were assessed using EdU analysis, and the EdU/DAPI ratio was calculated for five random visual fields (A549 group, *n* = 5 per group, two-way ANOVA, *F*_1, 16_ = 216.6, *P* < 0.0001, Šídák’s test, A549 scr-shRNA vs A549 Gαi1/3-DshRNA, *P* < 0.0001, A549 scr-shRNA vs A549 scr-shRNA treated with IL-11, *P* < 0.0001, A549 Gαi1/3-DshRNA vs A549 Gαi1/3-DshRNA treated with IL-11, *P* = 0.9986; H1299 group, *n* = 5 per group, two-way ANOVA, *F*_1, 16_ = 42.02, *P* < 0.0001, Šídák’s test, H1299 scr-shRNA vs H1299 Gαi1/3-DshRNA, *P* < 0.0001, H1299 scr-shRNA vs H1299 scr-shRNA treated with IL-11, *P* < 0.0001, H1299 Gαi1/3-DshRNA vs H1299 Gαi1/3-DshRNA treated with IL-11, *P* > 0.9999) (**C**). The abovementioned cell groups were treated with or without 50 ng/ml IL-11 for 24 h, and their migration (**D**) and invasion (**E**) abilities were assessed using the transwell assay. After crystal violet staining, the number of cells in five random visual fields was counted. **D** was analyzed using a two-way ANOVA (A549 group, *n* = 5 per group, two-way ANOVA, *F*_1, 16_ = 12.43, *P* = 0.0028, Šídák’s test, A549 scr-shRNA vs A549 Gαi1/3-DshRNA, *P* < 0.0001, A549 scr-shRNA vs A549 scr-shRNA treated with IL-11, *P* = 0.0004, A549 Gαi1/3-DshRNA vs A549 Gαi1/3-DshRNA treated with IL-11, *P* = 0.9993; H1299 group, n = 5 per group, two-way ANOVA, *F*_1, 16_ = 23.27, *P* = 0.0002, Šídák’s test, H1299 scr-shRNA vs H1299 Gαi1/3-DshRNA, *P* < 0.0001, H1299 scr-shRNA vs H1299 scr-shRNA treated with IL-11, *P* < 0.0001, H1299 Gαi1/3-DshRNA vs H1299 Gαi1/3-DshRNA treated with IL-11, *P* = 0.5105). Figure **E** was analyzed using a two-way ANOVA (A549 group, n = 5 per group, two-way ANOVA, *F*_1, 16_ = 38.50, *P* < 0.0001, Šídák’s test, A549 scr-shRNA vs A549 Gαi1/3-DshRNA, *P* < 0.0001, A549 scr-shRNA vs A549 scr-shRNA treated with IL-11, *P* < 0.0001, A549 Gαi1/3-DshRNA vs A549 Gαi1/3-DshRNA treated with IL-11, *P* = 0.6989; H1299 group, n = 5 per group, two-way ANOVA, *F*_1, 16_ = 14.96, *P* = 0.0014, Šídák’s test, H1299 scr-shRNA vs H1299 Gαi1/3-DshRNA, *P* < 0.0001, H1299 scr-shRNA vs H1299 scr-shRNA treated with IL-11, *P* < 0.0001, H1299 Gαi1/3-DshRNA vs H1299 Gαi1/3-DshRNA treated with IL-11, *P* = 0.9659). *P < 0.05. “N.S.” indicates *P* > 0.05. Scale bar = 100 μm.

Previous studies have demonstrated that GP130, Gαi1/3, and Gab1 can bind to each other to form complexes in the LUAD A549 cell line. Therefore, we next studied the functions of Gαi1 and Gαi3 proteins in LUAD. We used lentiviruses carrying Gαi1 shRNA and Gαi3 shRNA to interfere with the expression of Gαi1 and Gαi3 in A549 and H1299 cells. Stable cancer cells (A549 and H1299) bearing Gαi1/3 DshRNA were identified using puromycin screening. The mRNA levels of *Gαi1* and *Gαi3* in A549 (SFig. 3A) and H1299 (SFig. 3C) cells were found to be significantly decreased by Gαi1/3 DshRNA, but the mRNA levels of *Gαi2* were not significantly changed, as determined by qRT-PCR. Western blotting verified that the expression levels of Gαi1 and Gαi3 proteins in A549 (SFig. 3B) and H1299 (SFig. 3D) cells were significantly reduced by Gαi1/3 DshRNA, while the Gαi2 protein expression was not significantly changed. Next, A549 containing scr-shRNA (scramble control shRNA) and Gαi1/3-DshRNA were treated with 50 ng/ml recombinant human IL-11 protein, and activation of downstream IL-11 signals was detected by western blotting. The activation levels of Gab1, Jak2-STAT3, Akt-mTOR, and Erk1/2 in A549 containing Gαi1/3-DshRNA were found to be significantly decreased after IL-11 treatment compared with cells containing scr-shRNA (Fig. 6A). Similarly, we found that interference with the expression of Gαi1 and Gαi3 in H1299 cells significantly inhibited the activation of IL-11-induced signaling pathways (Fig. 6B). Moreover, we found through EdU experiments, that the proliferation abilities of A549 and H1299 cells were significantly inhibited after interfering Gαi1/3, and the decreased expression of Gαi1/3 significantly inhibited IL-11-induced cell proliferation (Fig. 6C). Through transwell assays, the migration and invasion abilities of A549 and H1299 cells were found to be inhibited by Gαi1/3 DshRNA. In addition, IL-11 was found to significantly induce LUAD cell migration (Fig. 6D) and invasion (Fig. 6E), and Gαi1/3 DshRNA was found to reverse this effect. These results demonstrated that silencing Gαi1/3 inhibits IL-11-induced cell proliferation, migration, and invasion in LUAD cells.

### Overexpression of Gαi1 and Gαi3 could promote the proliferation, migration, and invasion of LUAD cells in vitro and enhance the cancer-promoting ability of IL-11

**Fig. 7:**
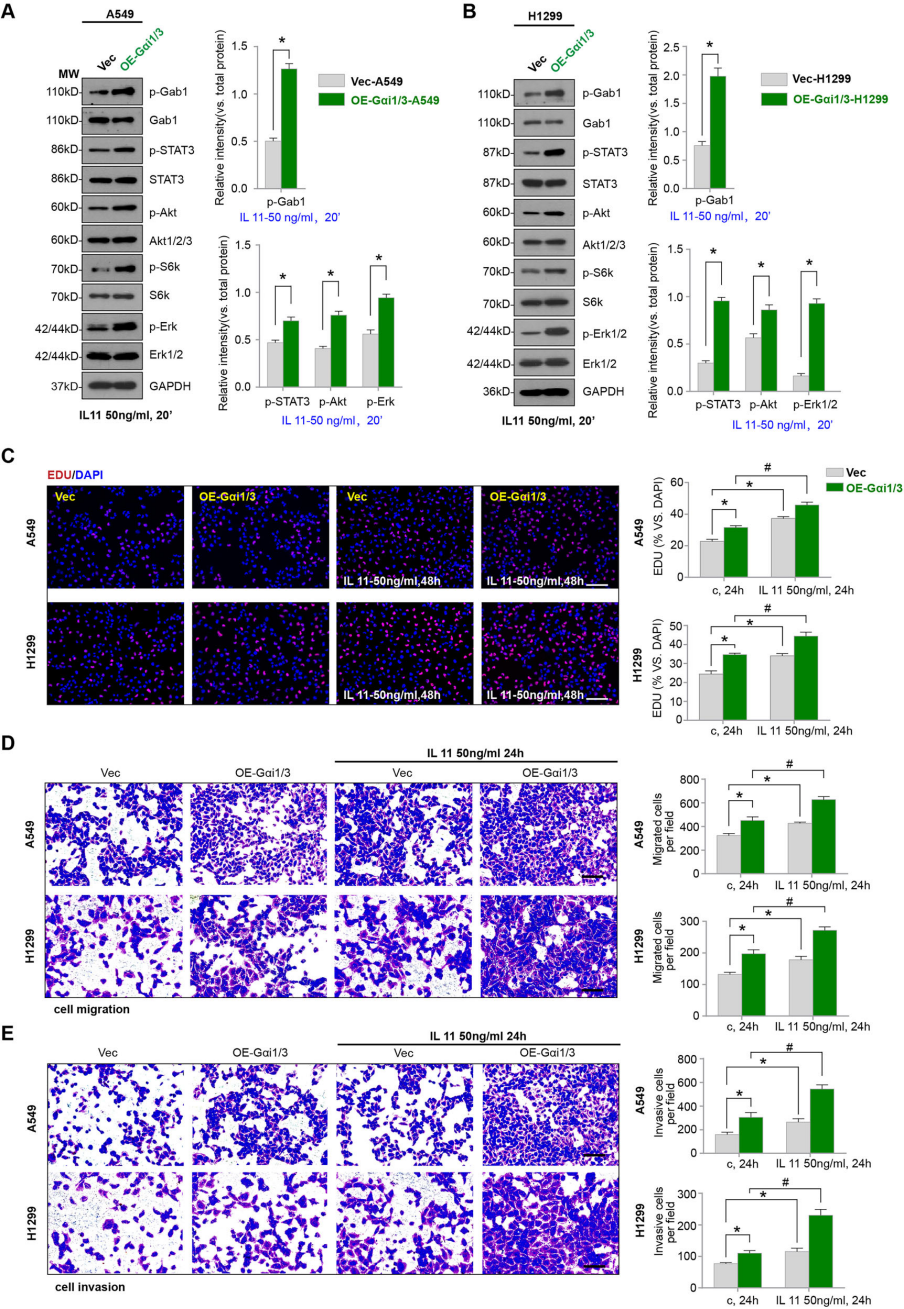
Overexpression of Gαi1 and Gαi3 could promote the proliferation, migration, and invasion abilities of LUAD cells in vitro and enhance the cancer-promoting ability of IL-11. Vec and OE-Gαi1/3 A549 (**A**) or H1299 (**B**) cells were treated with 50 ng/ml IL-11 for 20 min, and the expression levels of the listed proteins were then assessed using western blotting. After treatment with or without 50 ng/ml IL-11 for 48 h, the proliferation abilities of the abovementioned cell groups were assessed using EdU analysis, and the EdU/DAPI ratio was calculated for five random visual fields (A549 group, *n* = 5 per group, two-way ANOVA, *F*_1, 16_ = 0.03185, *P* = 0.8606, Šídák’s test, A549 vec vs A549 OE-Gαi1/3, *P* < 0.0001, A549 vec vs A549 vec treated with IL-11, *P* < 0.0001, A549 OE-Gαi1/3 vs A549 OE-Gαi1/3 treated with IL-11, *P* < 0.0001; H1299 group, n = 5 per group, two-way ANOVA, *F*_1, 16_ = 0.003505, *P* = 0.9535, Šídák’s test, H1299 vec vs H1299 OE-Gαi1/3, *P* < 0.0001, H1299 vec vs H1299 vec treated with IL-11, *P* < 0.0001, H1299 OE-Gαi1/3 vs H1299 OE-Gαi1/3 treated with IL-11, *P* < 0.0001) (**C**). The abovementioned cell groups were treated with or without 50 ng/ml IL-11 for 24 h, and their migration (**D**) and invasion (**E**) abilities were assessed using the transwell assay. After crystal violet staining, the number of cells in five random visual fields was counted. **D** was analyzed using a two-way ANOVA (A549 group, *n* = 5 per group, two-way ANOVA, *F*_1, 16_ = 13.88, *P* = 0.0018, Šídák’s test, A549 vec vs A549 OE-Gαi1/3, *P* < 0.0001, A549 vec vs A549 vec treated with IL-11, *P* < 0.0001, A549 OE-Gαi1/3 vs A549 OE-Gαi1/3 treated with IL-11, *P* < 0.0001; H1299 group, n = 5 per group, two-way ANOVA, *F*_1, 16_ = 8.493, *P* = 0.0101, Šídák’s test, H1299 vec vs H1299 OE-Gαi1/3, *P* < 0.0001, H1299 vec vs H1299 vec treated with IL-11, *P* < 0.0001, H1299 OE-Gαi1/3 vs H1299 OE-Gαi1/3 treated with IL-11, *P* < 0.0001). **E** was analyzed using a two-way ANOVA (A549 group, *n* = 5 per group, two-way ANOVA, *F*_1, 16_ = 22.08, *P* = 0.0002, Šídák’s test, A549 vec vs A549 OE-Gαi1/3, *P* < 0.0001, A549 vec vs A549 vec treated with IL-11, *P* = 0.0005, A549 OE-Gαi1/3 vs A549 OE-Gαi1/3 treated with IL-11, *P* < 0.0001; H1299 group, *n* = 5 per group, two-way ANOVA, *F*_1, 16_ = 64.81, *P* < 0.0001, Šídák’s test, H1299 vec vs H1299 OE-Gαi1/3, *P* = 0.0022, H1299 vec vs H1299 vec treated with IL-11, *P* = 0.0005, H1299 OE-Gαi1/3 vs H1299 OE-Gαi1/3 treated with IL-11, *P* < 0.0001). *P < 0.05. #*P* < 0.05. Scale bar = 100 μm.

Lentiviral constructs encoding Gαi1 cDNA and Gαi3 cDNA were transduced into A549 and H1299 cells (A549/H1299 OE-Gαi1/3 cells). Cells with stable Gαi1/3 expression were identified using puromycin screening. The mRNA expression levels of *Gαi1/3* in A549 OE-Gαi1/3 (SFig. 3E) and H1299 OE-Gαi1/3 (SFig. 3G) cells were found to be up-regulated by qRT-PCR. Western blotting verified that the protein expression levels of Gαi1 and Gαi3 in A549 OE-Gαi1/3 (SFig. 3F) and H1299 OE-Gαi1/3 (SFig. 3H) cells were up-regulated. We subsequently found that overexpression of Gαi1/3 in A549 (Fig. 7A) and H1299 (Fig. 7B) cells enhanced the IL-11-induced activation of Gab1, Jak2-STAT3, Akt-mTOR, and Erk1/2. Results of the EdU analysis showed that Gαi1/3 overexpression promoted the proliferation of A549 and H1299 cells and enhanced the IL-11-induced proliferation of A549 and H1299 cells (Fig. 7C). In addition, transwell assay results showed that overexpression of Gαi1/3 could also improve the migration and invasion abilities of LUAD cells and promote IL-11-induced migration (Fig. 7D) and invasion (Fig. 7E) of LUAD cells to a certain extent. These findings indicated that the overexpression of Gαi1/3 could enhance the IL-11-induced proliferation, migration, and invasion of LUAD cells in vitro.

### Depletion of Gαi1 and Gαi3 inhibited the growth and metastasis of subcutaneous LUAD xenografts in vivo

**Fig. 8:**
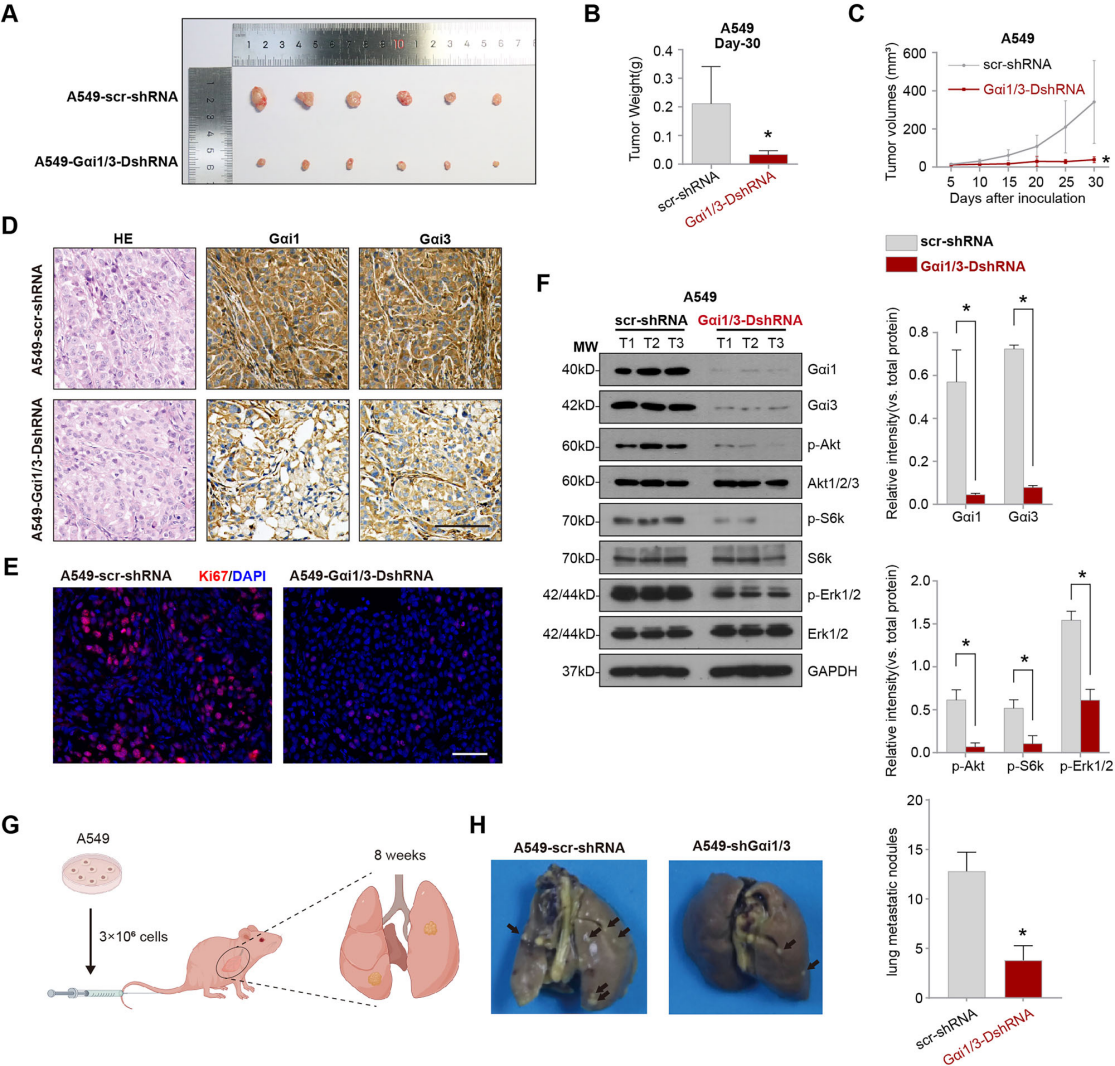
Depletion of Gαi1 and Gαi3 inhibited the in vivo growth of subcutaneous LUAD xenografts. A nude mouse LUAD model was established by subcutaneous injection of A549 transfected with Gαi1/3 DshRNA or scr-shRNA lentivirus. After 30 days, the established tumors were photoed (**A**) and weighed (**B**) (from six mice in each group). Tumor volumes were recorded every five days (**C**). HE and IHC results of sections of xenografts (**D**). Ki67 immunofluorescence staining of sections of xenografts (**E**). Western Blotting was used to assess the expression levels of listed proteins in the graft-induced tumors (**F**). Flowchart of the lung metastasis models (**G**). Scr-shRNA and shGαi1/3 A549 cells were injected into the tail vein of nude mice, respectively, and the lungs were removed at the end of the experiment to take photographs and record the lung metastatic nodules (**H**) (from five mice in each group). *P < 0.05. Scale bar = 100 μm.

Our in vitro experiments showed that silencing Gαi1/3 inhibited the proliferation, migration, and invasion of A549 and H1299 cells. Simultaneously, in shGαi1/3 A549 cells, we observed an increased proportion of TUNEL-positive nuclei (SFig. 4A). Furthermore, western blotting analysis of Gαi1/3-knockdown A549 cells revealed significantly increased cleaved forms of caspase-3 and caspase-9 (SFig. 4B). Collectively, these findings confirmed that apoptosis activation was associated with Gαi1/3 deficiency. Therefore, we next investigated the effect of Gαi1 and Gαi3 proteins on the growth of LUAD cells in vivo. Equal numbers of A549 cells transfected with Gαi1/3 shRNA or scramble control shRNA were injected subcutaneously into the right axilla of nude mice (six mice per group). When the grafts were removed 30 days later, we found that the Tumors expressing Gαi1/3 shRNA were significantly lighter and smaller than those expressing scramble control shRNA (Fig. 8A–C). After subcutaneous cell injection, subcutaneous tumor volume was measured with vernier calipers every 5 days, and the growth of subcutaneous tumors in the Gαi1/3-DshRNA group was found to be significantly inhibited (Fig. 8C). IHC studies confirmed Gαi1 and Gαi3 protein silencing in Gαi1/3-DshRNA xenograft slides (Fig. 8D). Ki67 immunofluorescence staining was performed on sections of the subcutaneous tumors. The results showed that the expression of Ki67, a proliferative marker, was significantly lower in the grafts with Gαi1/3 silencing (Fig. 8E). Moreover, the phosphorylation levels of Akt, S6k, and Erk1/2 were significantly decreased in Gαi1/3 shRNA-expressing tumors (Fig. 8F). These results suggested that Gαi1/3 affected the growth of LUAD in vivo by activating the Akt-mTOR and Erk1/2 signaling pathways.

To assess the effect of Gαi1/3 on LUAD cell metastasis in vivo, we established a caudal vein injection model. We injected scr-shRNA or shGαi1/3 A549 cells into the tail veins of nude mice (five mice per group). After 8 weeks, the mice were euthanized, their lungs were removed, fixed in Bouin’s solution, and subsequently photographed to visualize metastatic nodules (Fig. 8G, H). Results showed that Gαi1/3 silencing led to a significant reduction in the number of lung metastatic nodules compared with the scr-shRNA group (Fig. 8H). In conclusion, our findings suggested that Gαi1/3 were required for the growth and metastasis of LUAD.

### Gαi1 expression levels were upregulated in LUAD and correlated with poor prognosis

**Fig. 9:**
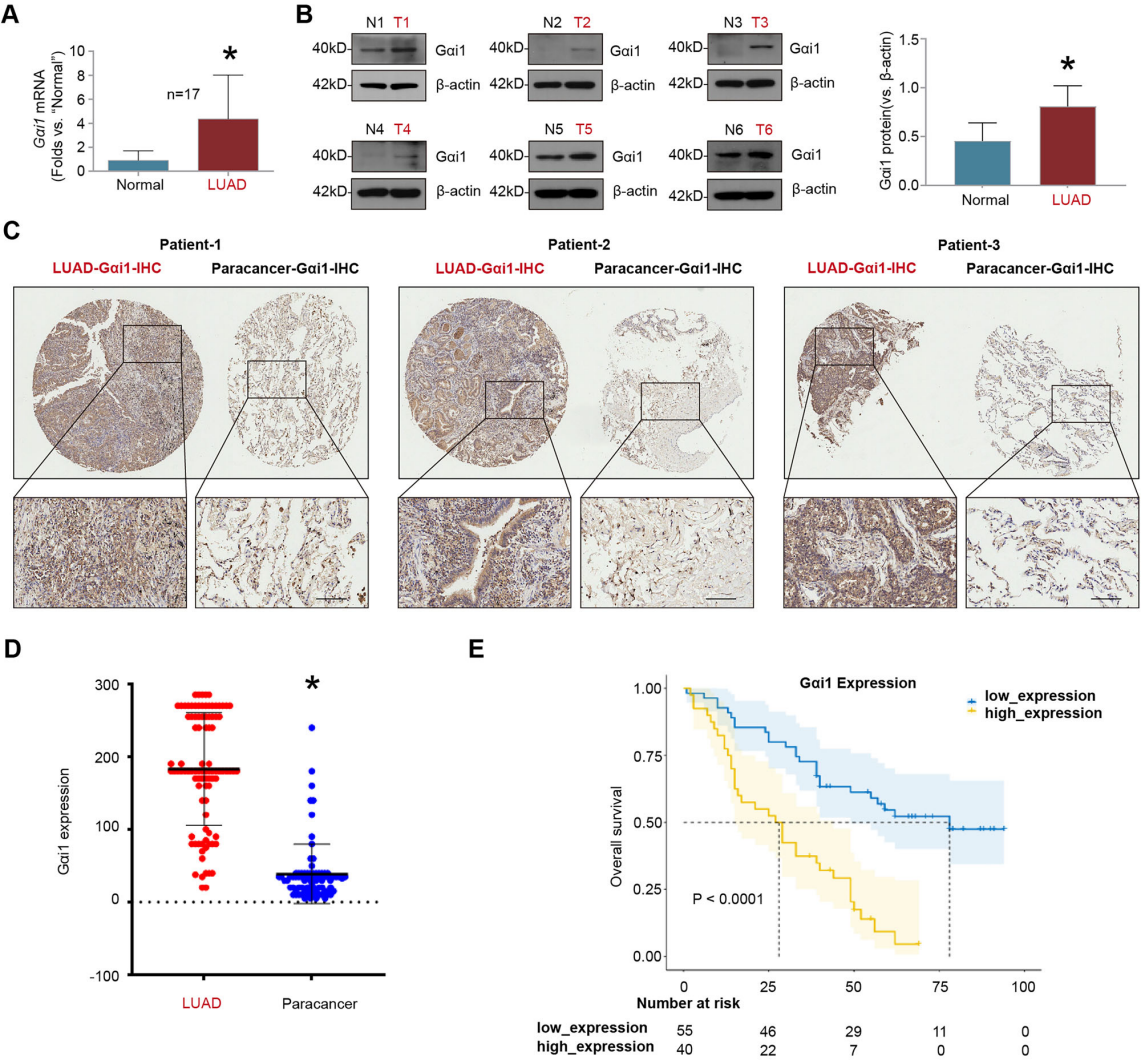
Gαi1 expression levels were upregulated in LUAD and correlated with poor patient prognosis. *Gαi1* mRNA expression levels in human LUAD tumor tissues (“LUAD”) and paracancerous tissues (“Normal”) were tested using qRT-PCR (**A**). Gαi1 proteins expression levels in human LUAD tumor tissues (“T”) and paracancerous tissues (“N”) were tested using western blotting (**B**). Representative tissue microarray IHC images showing the expression levels of Gαi1 in LUAD tumor tissues and adjacent normal tissues (**C**). IHC scores of Gαi1 in LUAD tumor tissues and adjacent normal tissues (**D**). The Kaplan-Meier overall survival curve according to Gαi1 IHC scores in LUAD patients is shown (**E**). *P < 0.05. Scale bar = 100 µm.

We have previously demonstrated that *Gαi3* mRNA and protein levels were upregulated in LUAD and negatively correlated with favorable patient prognosis [21]. To verify whether *Gαi1* was oncogene, we performed qRT-PCR analysis on 17 LUAD tissues and paired normal lung tissues. We found that *Gαi1* was highly expressed in LUAD tissues (Fig. 9A). Western blotting also demonstrated that Gαi1 was highly expressed in LUAD tissues (Fig. 9B). To further investigate the relationship between the expression levels of Gαi1 and the prognosis of LUAD patients, IHC was performed on LUAD tissue microarrays to detect the expression of Gαi1 (SFig. 5). Gαi1 protein expression was significantly higher in LUAD tissues (“T”) than in normal lung tissues (“N”). Representative Gαi1 IHC images of three representative patients (“Patient-1” to “Patient-3”) further showed that Gαi1 protein was upregulated in LUAD tissues (Fig. 9C). The tissue microarray IHC results showed that the Gαi1 proteins were mainly located in the cytoplasm of LUAD cells and adjacent tissue cells. We compared the IHC scores of LUAD and adjacent tissues using the Mann-Whitney test and found that Gαi1 protein expression levels were higher in LUAD tissues than those in the adjacent tissues (Fig. 9D). Patients were stratified into Gαi1-high and -low subgroups using a median IHC score cutoff. The overall survival time was found to be shorter in patients with high Gαi1 expression levels (Fig. 9E). Together, these findings suggested that high expression levels of Gαi1 in LUAD tissues was associated with poor patient prognosis.

Finally, we analyzed the relationship between mRNA levels of *IL-11*, *Gαi1*, and *Gαi3* in the TCGA database and clinical case characteristics of LUAD patients (Supplementary Tables 5-7). Results indicate that *IL-11* expression correlates with patient age, pathological stage, pathological T stage, smoking status, and overall survival (Supplementary Table 5). Among *Gαi1* and *Gαi3*, only *Gαi3* was associated with the patient’s pathological T stage and pathological N stage (Supplementary Tables 6, 7). Additionally, we analyzed the relationship between *IL-11* expression and *Gαi1* and *Gαi3* in LUAD patients. The expression of *IL-11* was positively correlated with both *Gαi1* and *Gαi3* (SFig. 6A, B). Based on our preliminary experimental results, we further concluded that targeted therapy against downstream Gαi1 and Gαi3 could be applied to lung adenocarcinoma patients with high IL-11 expression.

## Discussion

IL-11 has a wide range of origins in lung tumors, including tumor cells, fibroblasts, and epithelial cells [13]. Here we similarly found that IL-11 was predominantly expressed in fibroblasts and epithelial cells in the lung cancer microenvironment by analyzing single-cell sequencing data. These results suggest that in the tumor microenvironment IL-11 may affect tumor cells by autocrine or paracrine means, which in turn promotes the progression of lung cancer by activating relevant signaling pathways.

There has been increasing evidence that IL-11 is a potential biomarker in NSCLC patients [32]. In addition, IL-11RA is variably expressed in NSCLC and has been targeted to reduce tumor growth in preclinical studies [33]. Increased IL-11 expression in cisplatin-stimulated cancer-associated fibroblasts (CAFs) was associated with drug resistance in lung cancer patients [13]. Hypoxia is a hallmark of cancer and is associated with poor prognosis [34]. Hypoxic environments similarly lead to upregulation of IL-11 expression in NSCLC cells [35]. In lung cancer brain metastases, IL-11 secreted by astrocytes promoted the upregulation of PDL1 in tumor cells through activation of the EGFR and GP130 pathways, which in turn triggered immune escape [36]. IL-11 was reported to be associated with a variety of other malignancies, which indicates that it can act as a direct or indirect therapeutic target for these tumors [37]. Christina Heichler et al. found that CAFs promote tumor development and are associated with poor prognosis through IL-6/IL-11-activated STAT3 in colorectal cancer [38]. In addition, some studies have shown that IL-11 can inhibit CD4 + T cell-mediated antitumor responses in the tumor microenvironment and thereby promote tumor growth [39, 40].

Here we further confirmed the high expression of IL-11 in LUAD by bioinformatics analysis and immunofluorescence experiments, and its overexpression correlates with poor OS, DSS and PFI of the patients. Through GSEA enrichment analysis, IL-11 was found to be correlated with multiple HALLMARK pathways, which better interpreted the ability of IL-11 to promote the progress of LUAD. We also analyzed the correlation between the expression of IL-11 and the clinicopathological information of LUAD patients in the TCGA database, and found that the expression of IL-11 is related to smoking, but the specific mechanism of smoking leading to the expression of IL-11 remains unclear. In conclusion, inhibition of IL-11 signaling pathway transduction is an effective strategy for the treatment of LUAD.

**Fig. 10:**
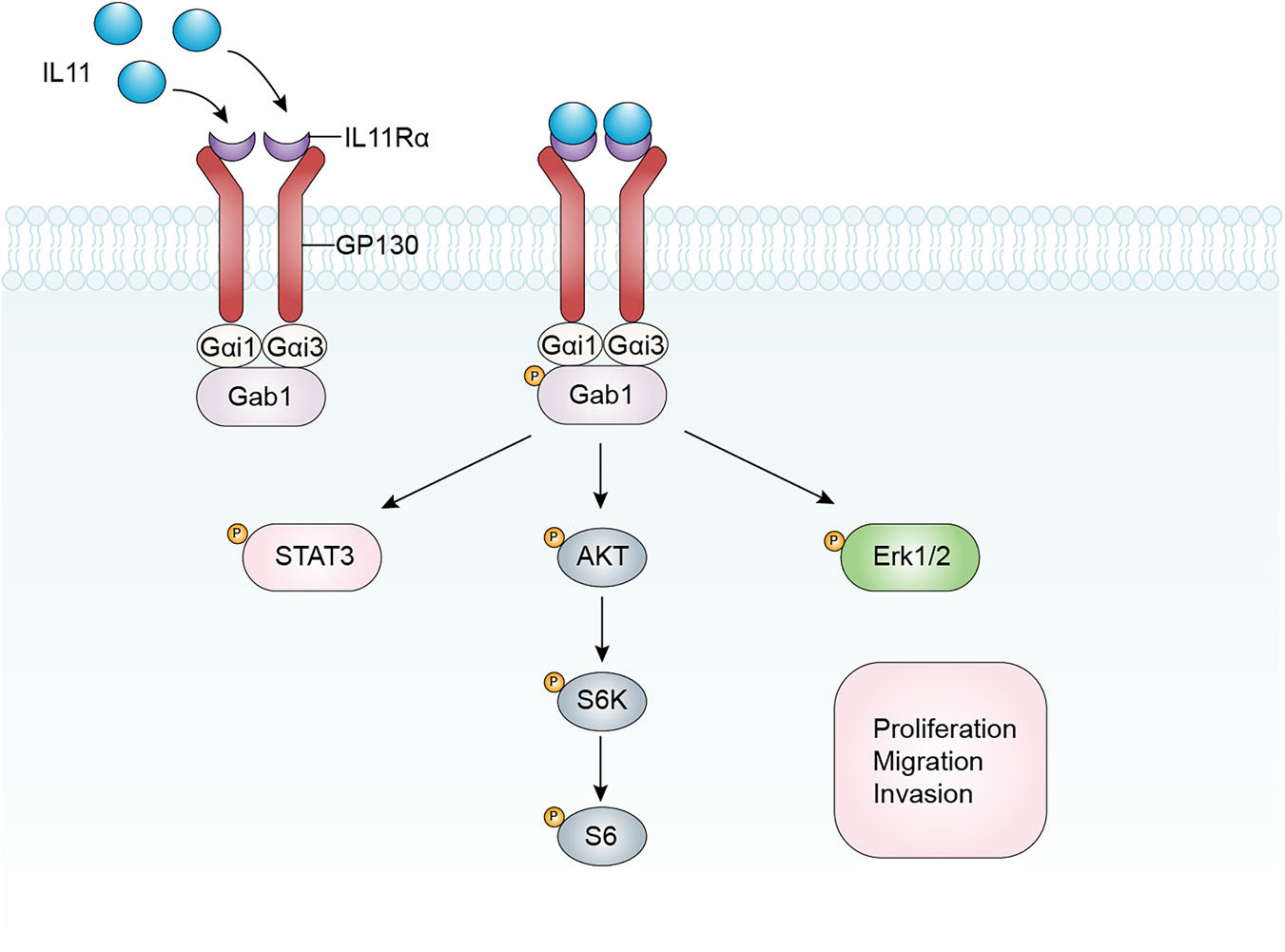
The proposed signaling mechanism derived from this study.

We have previously shown that Gαi1 and Gαi3 proteins are required for a variety of RTKs and can mediate the signaling of multiple growth factors, such as stem cell factor (CSF) [16], vascular endothelial growth factor (VEGF) [41], keratinocyte growth factor (KGF) [30], and brain-derived neurotrophic factor (BDNF) [42]. In the present study, we further identified Gαi1/3 as a key protein in IL-11 signaling. A diagram of the IL-11 signaling mechanism in this study is shown in Fig. 10. Gαi1 SKO and Gαi3 SKO in MEFs partially inhibited the IL-11-induced activation of Jak2-STAT3, PI3K-Akt, and Erk-MAPK, and this inhibition was more pronounced in response to Gαi1/3 DKO. Silencing of Gαi1 and Gαi3 by shRNA and CRISPR-Cas9 similarly inhibited IL-11 signaling in MEFs. Re-expression of Gαi1 and Gαi3 in Gαi1/3 DKO MEFs rescued IL-11 signaling. Moreover, ectopic expression of Gαi1 and Gαi3 in MEFs enhanced IL-11 signaling. Similarly, in A549 and H1299 cells, Gαi1/3 shRNA inhibited the IL-11-induced activation of Jak2-STAT3, PI3K-Akt, and Erk-MAPK, but ectopic Gαi1/3 overexpression enhanced the IL-11-induced activation of Jak2-STAT3, PI3K-Akt, and Erk-MAPK. In the present study, we found that Gαi1/3 can form a complex with GP130 and p-Gab1 to mediate IL-11 signaling through CO-IP assays. Post-translational modifications of C18:0 (stearate) and C18:1 (oleate) have been reported to attenuate the ability of Gαi to activate the EGFR signaling pathway through the recruitment of the junction protein Gab1, and it may be able to mediate IL-11-induced signaling in the same way to treat LUAD [43]. IL-11 not only promotes the growth of tumor cells but also promotes cardiovascular fibrosis and idiopathic pulmonary fibrosis, which are also involved in other human diseases [7, 44, 45]. Blocking the expression of Gαi1/3 may inhibit the side effects of IL-11 overexpression.

Previous studies have found that neutralizing antibodies targeting IL-11RA exhibit significant anti-tumor efficacy in patient-derived xenograft models [46]. Here, our research reveals a core signaling module composed of GP130, Gαi1/3, and Gab1, which provides a theoretical basis for developing novel targeted therapies for LUAD. Based on the findings of this study, we propose a highly promising therapeutic direction for the future: the development of highly selective small-molecule inhibitors or neutralizing antibodies targeting Gαi1/3. Given the central role of Gαi1/3 in mediating IL-11 signaling, such drugs are expected to directly block key downstream oncogenic signal transduction of this pathway. More importantly, considering that Gαi1/3 is highly expressed in LUAD and associated with poor prognosis, targeting Gαi1/3 may become a precise personalized treatment strategy, particularly suitable for LUAD patient populations with high IL-11 expression. Combining Gαi1/3-targeted drugs with existing standard therapies may produce synergistic anti-tumor effects. First, combination with immune checkpoint inhibitors warrants in-depth exploration. This study and previous work both indicate that IL-11 has an immunosuppressive effect on anti-tumor immunity [39, 40]. Therefore, inhibiting Gαi1/3 is expected to reverse IL-11-mediated immune suppression and remodel the tumor microenvironment, potentially converting “cold tumors” into “hot tumors”, thereby enhancing the efficacy of anti-PD-1/PD-L1 antibodies [46]. Second, Gαi1/3 simultaneously activates multiple pro-survival pathways such as Akt-mTOR, Erk, and STAT3, suggesting that combining its inhibitors with downstream targeted drugs could achieve a “vertical blockade” of key oncogenic pathways, potentially effectively preventing or overcoming the common issues of feedback activation and drug resistance following single-pathway inhibition.

Gαi1/3 specifically mediates the downstream signaling of IL-11 (Akt, Erk, STAT3) by binding to the GP130 receptor subunit and recruiting Gab1. GP130 is a common signal-transducing receptor for the IL-6 family of cytokines [47]. In addition to IL-11, this family also includes IL-6, leukemia inhibitory factor (LIF), oncostatin M (OSM), ciliary neurotrophic factor (CNTF), cardiotrophin-1 (CT-1), and others. Therefore, inhibiting Gαi1/3 is highly likely to also affect the signaling of other cytokines that depend on GP130. Targeting Gαi1/3 may attenuate IL-6-induced pro-inflammatory and pro-tumorigenic signaling, affect the role of LIF in maintaining stem cell stemness and tumorigenesis, and interfere with OSM’s functions in inflammation and cancer progression [48]. Although they share GP130, different IL-6 family cytokines have distinct specific receptor alpha chains (such as IL-6Rα, IL-11Rα, LIFR), which may result in subtle differences in the conformation of the receptor complexes. Therefore, the extent of Gαi1/3’s contribution may vary among different cytokines. Furthermore, as a core member of G proteins, perturbation of Gαi1/3 affects the classical GPCR signaling network and its “crosstalk” with other growth factor RTK pathways, leading to complex intracellular signal reprogramming. Therefore, to successfully translate Gαi1/3 into a safe and effective therapeutic target, a thorough and prudent evaluation of its central role and potential impacts within the entire signaling network is essential.

The Gαi protein has been reported as a potential therapeutic target in various cancers [49, 50]. Liu et al. found that the transcription factor TCF7L2 binds to the promoter region of *Gαi3* and promotes the growth of pancreatic cancer by promoting the expression of *Gαi3* [51]. Whereas ZNF384 can act as a transcription factor for *Gαi1* [52]. We have previously demonstrated through multiple experiments that Gαi3 is highly expressed in lung adenocarcinoma and may serve as a potential target for lung adenocarcinoma therapy [21]. Further, qRT-PCR and western blotting showed that *Gαi1* mRNA and protein expression levels, respectively, were higher in LUAD tissues than in normal paracancerous tissues. Our findings are supported by the results of immunohistochemistry on tissue microarray IHC analyses. The immunohistochemistry scores for Gαi1 were higher in LUAD tissues than normal lung tissues, and patients with high Gαi1 IHC scores had a poorer prognosis. These results suggest that Gαi1, as well as Gαi3, is a potential therapeutic target for LUAD.

## Conclusion

The Gαi1/3-Gab1 complex is essential for IL-11 to mediate downstream STAT3, Akt-mTOR and Erk activation. The IL-11-GP130-Gαi1/3 signaling pathway is involved in the proliferation, migration, and invasion of LUAD, providing a new target for the molecular treatment of LUAD.

## Supplementary information

Supplementary Tables

Supplementary Figures

The uncropped blotting images of the study.

## Data Availability

The sequence data for both the A549 scr-shRNA cells and A549 shIL-11 cells produced in this study were deposited in the NCBI GEO repository with the accession code GSE317274.
