# Supplementary material for: Gαi1 and Gαi3 mediate IL-11-induced signal transduction and are potential therapeutic targets for LUAD
Source: Cell Death Dis. 2026 Apr 25;17(1):554. doi: 10.1038/s41419-026-08637-w (PMC13247079; doi:10.1038/s41419-026-08637-w)
Supplement: Supplementary file 2 — Supplementary Figures [file 41419_2026_8637_MOESM2_ESM.docx]

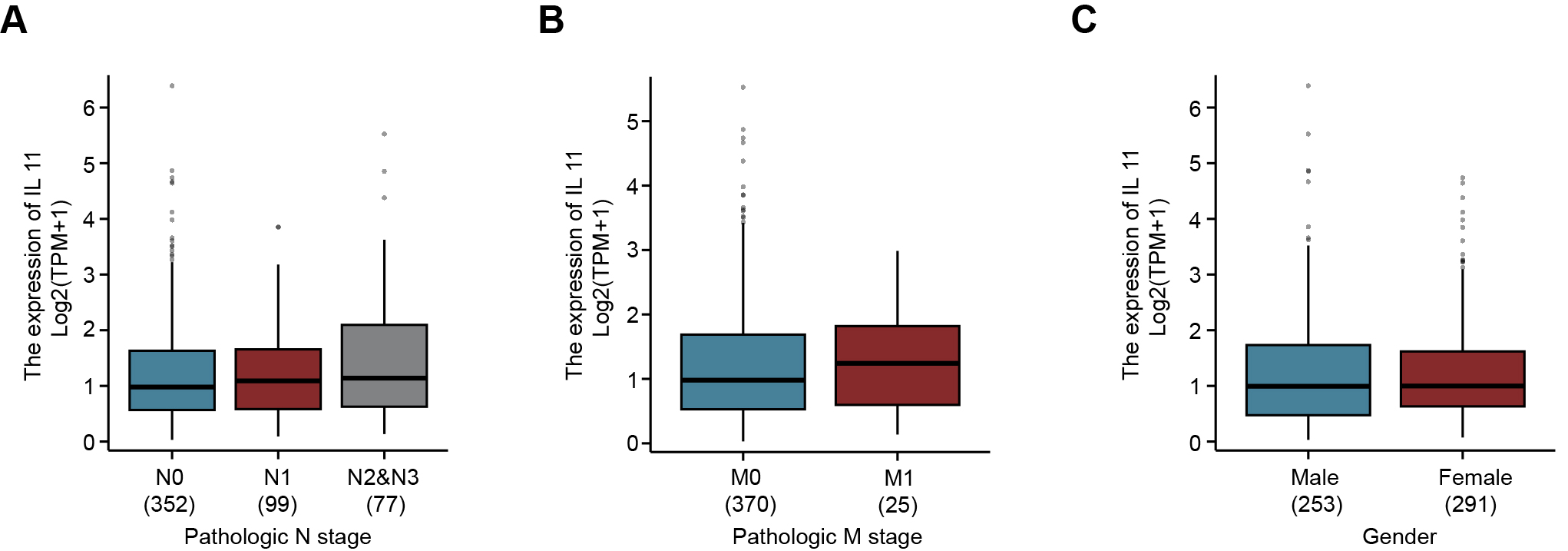


**Supplementary Figure 1**

**Correlation of *IL-11* expression in human LUAD tissues with key clinical parameters in patients.** Within the TCGA-LUAD cohort, *IL-11* expression in the specific LUAD patients with described clinical parameters were shown (**A–C**).


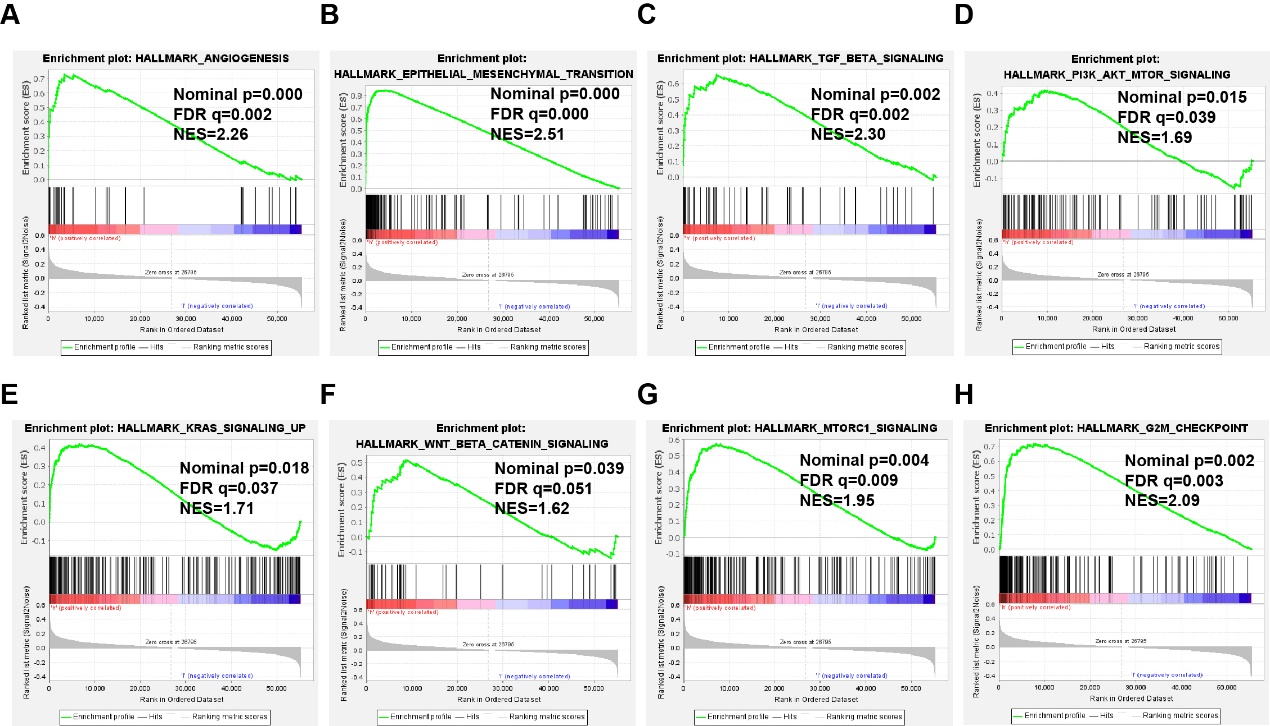


**Supplementary Figure 2**

***IL-11*-related GSEA enrichment analysis results.** Compared to low *IL-11* expressing LUAD patients, high *IL-11* expressing LUAD patients had significantly enriched expression of genes characterized by angiogenesis (**A**), epithelial mesenchymal transition (**B**), TGF-β signaling (**C**), PI3K-AKT-mTOR signaling (**D**), kras signaling (**E**), wnt-β-catenin signaling (**F**), mTORC1 signaling (**G**) and G2M checkpoint (**H**), as shown by GSEA.


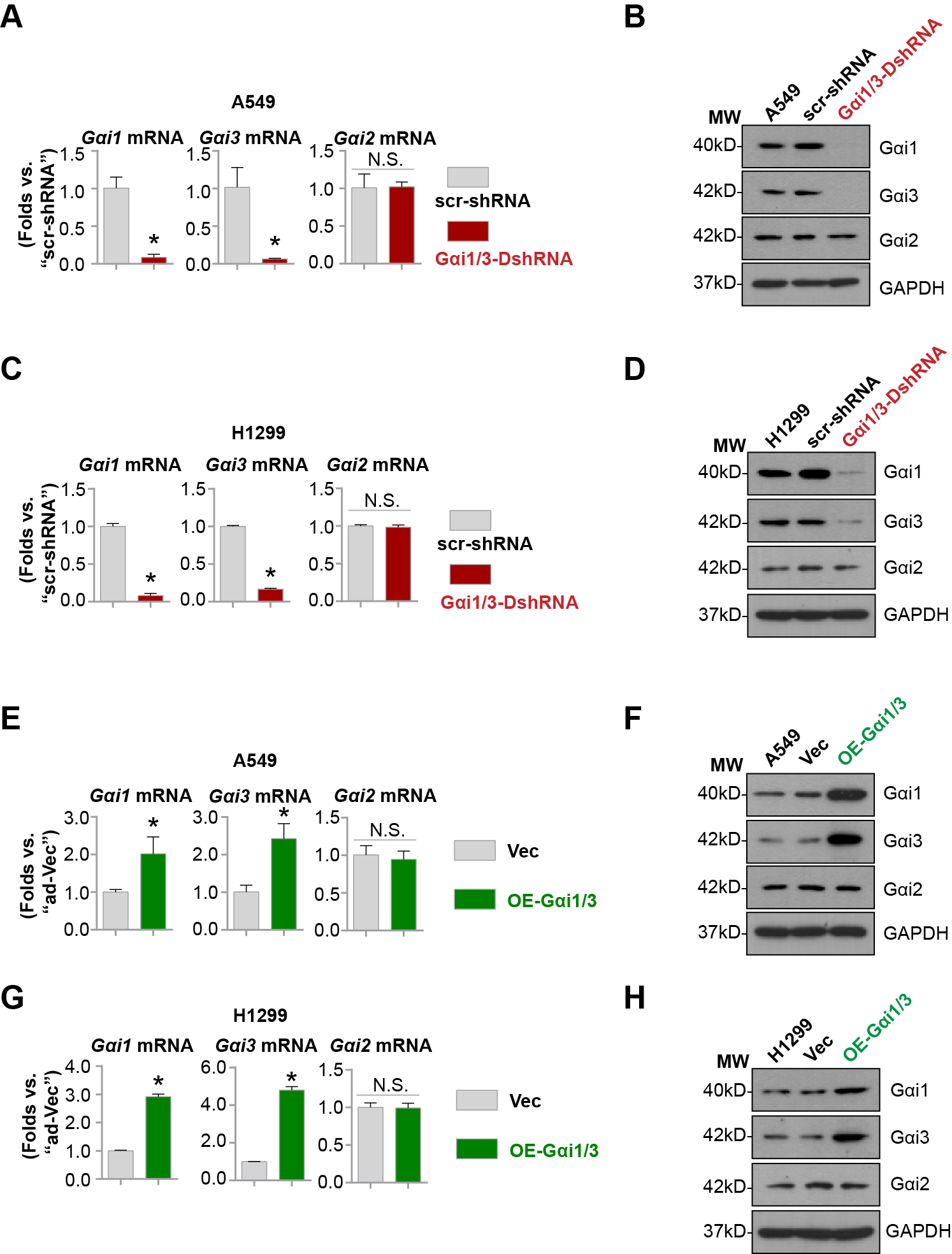


**Supplementary Figure 3**

**Construct LUAD cells with stable Gαi1/3 overexpression or knockdown.**

A549 cells were transfected with Gαi1/3 shRNA or scramble control shRNA and the expression levels of Gαi1, Gαi2, and Gαi3 were assessed using qRT-PCR (**A**) and western blotting (**B**). H1299 cells were transfected with Gαi1/3 shRNA or scramble control shRNA, and the expression levels of *Gαi1*, *Gαi2*, and *Gαi3* were assessed using qRT-PCR (**C**) and western blotting (**D**). A549 cells were transfected with an empty vector or lentiviral constructs encoding full length Gαi1 and Gαi3 cDNAs. The expression levels of *Gαi1*, *Gαi2*, and *Gαi3* were then assessed using qRT-PCR (**E**) and western blotting (**F**). H1299 cells were transfected with an empty vector or lentiviral constructs encoding full length Gαi1 and Gαi3 cDNAs. The expression levels of *Gαi1*, *Gαi2*, and *Gαi3* were then assessed using qRT-PCR (**G**) and western blotting (**H**). ****P*** < 0.05. “N.S.” indicates ***P*** > 0.05.


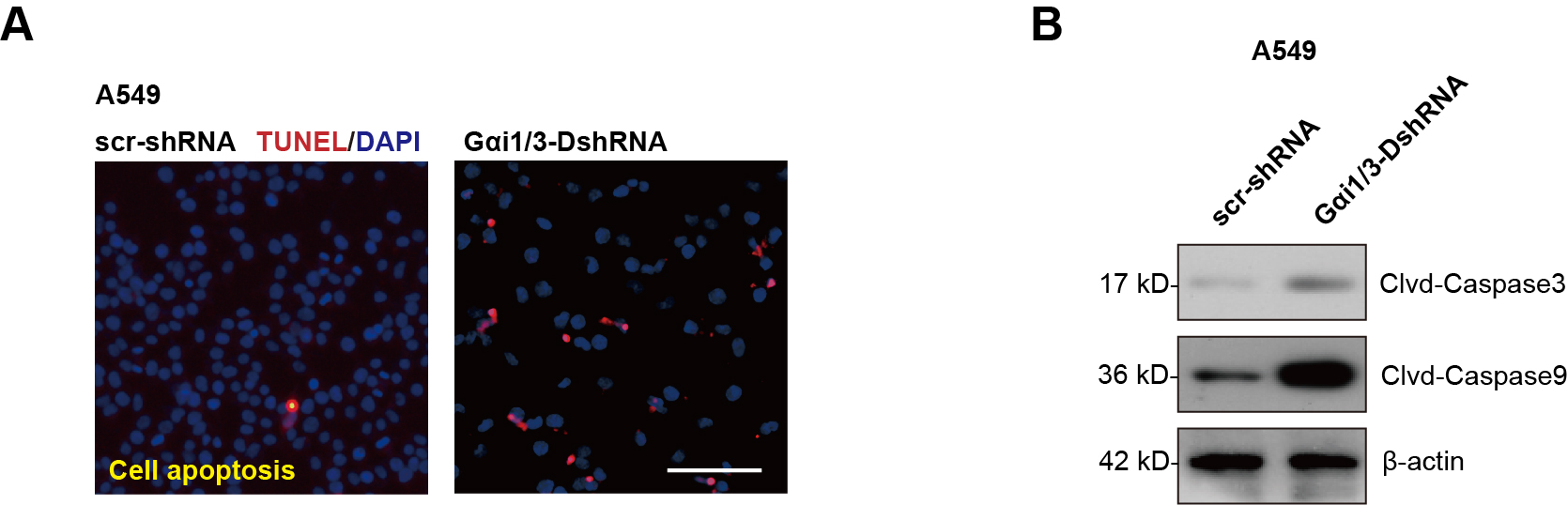


**Supplementary Figure 4**

**Apoptosis activation after Gαi1/3 depletion in LUAD cells.**

The level of apoptosis in the relevant cells was detected by TUNEL positive nuclear ratios (**A**) and caspase 3 and caspase 9 cleavage protein expression levels (**B**).


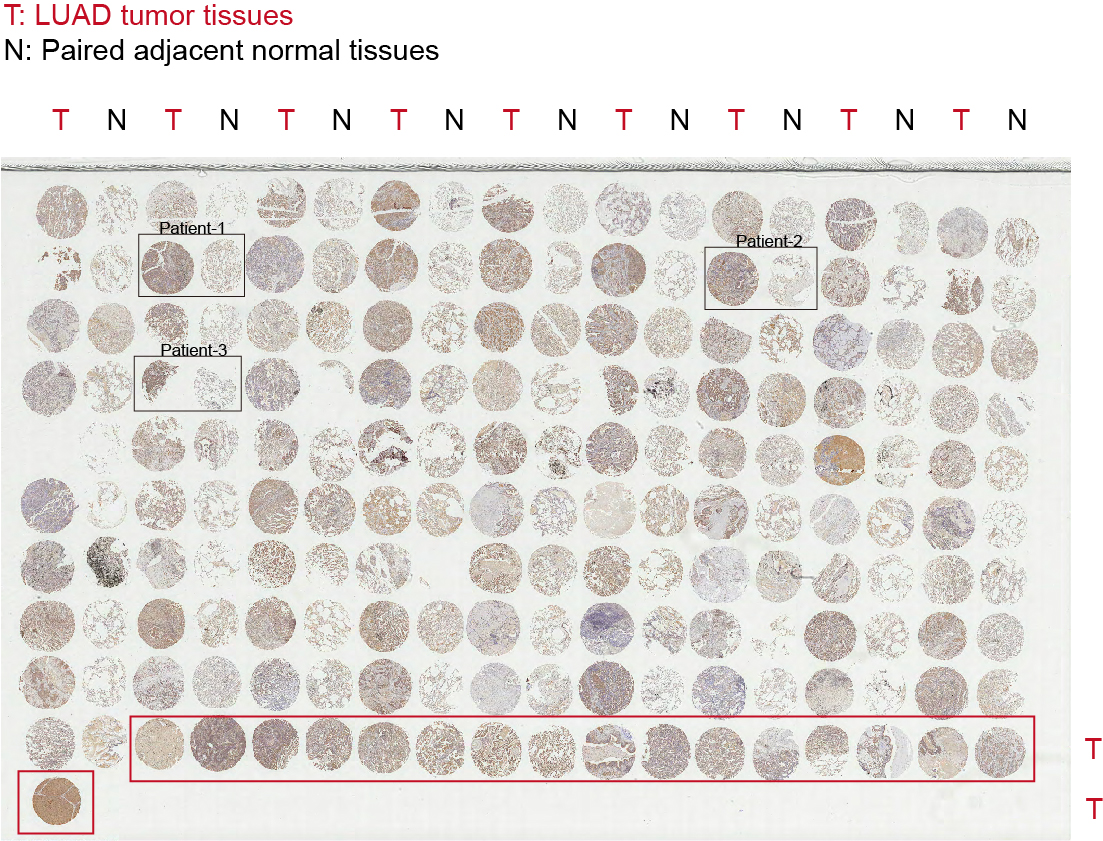


**Supplementary Figure 5**

**Lung adenocarcinoma microarray.** Immunohistochemical staining of Gαi1 in lung adenocarcinoma microarray.


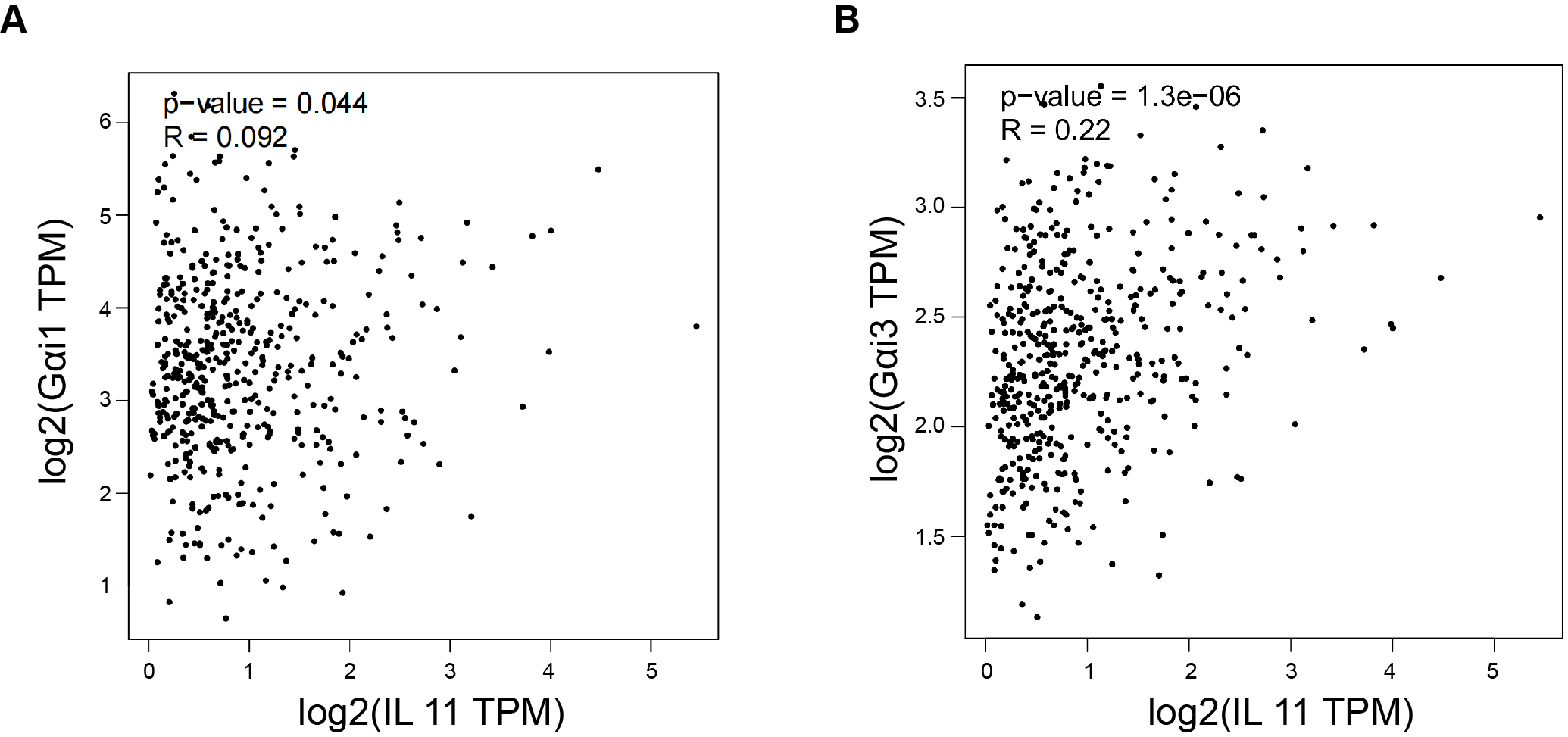


**Supplementary Figure 6**

**Correlation Analysis.** Use the GEPIA2 online platform (http://gepia2.cancer-pku.cn/#index) to analyze the correlation between IL11 expression with Gαi1 (**A**) and Gαi3 (**B**) expression in LUAD patients.
