# Supplementary material for: Gαi1 and Gαi3 mediate IL-11-induced signal transduction and are potential therapeutic targets for LUAD
Source: Cell Death Dis. 2026 Apr 25;17(1):554. doi: 10.1038/s41419-026-08637-w (PMC13247079; doi:10.1038/s41419-026-08637-w)

Figure 1E

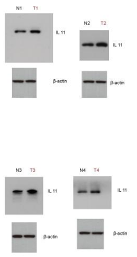

Figure 3A

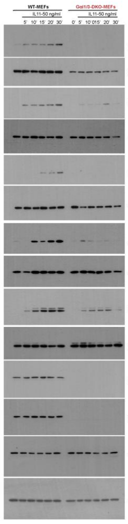

Figure 3B

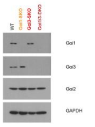

Figure 3C

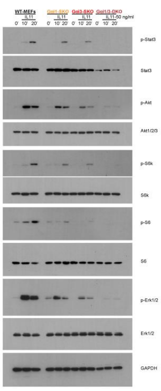

Figure 3D

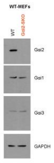

Figure 3E

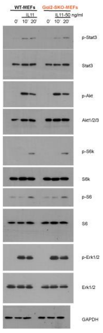

Figure 4A

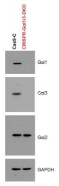

Figure 4B

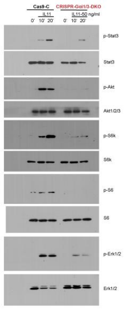

Figure 4C

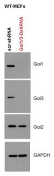

Figure 4D

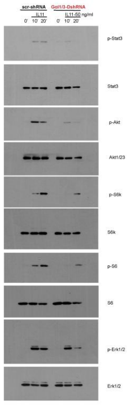

Figure 4E

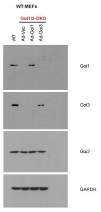

Figure 4F

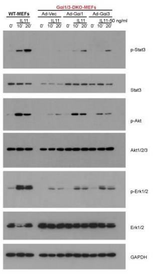

Figure 4G

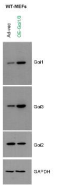

Figure 4H

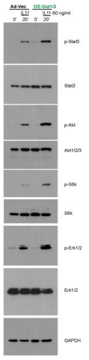

Figure 5G

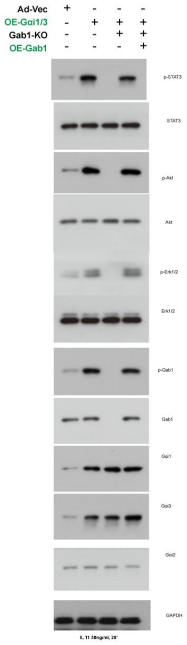

Figure 5H

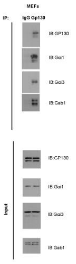

Figure 5I

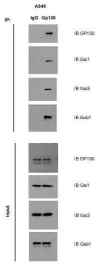

Figure 5A

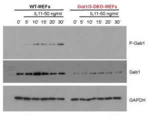

Figure 5B

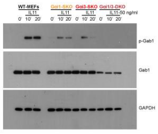

Figure 5C

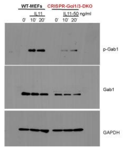

Figure 5D

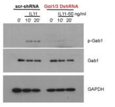

Figure 5E

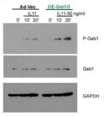

Figure 5F

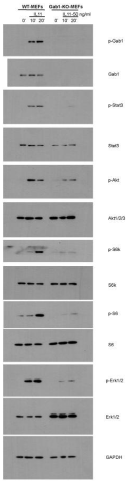

Figure 5M

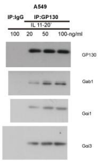

Figure 6A

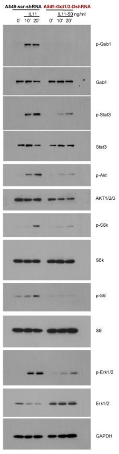

Figure 6B

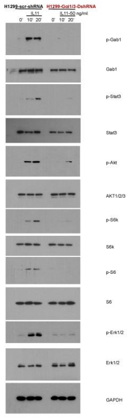

SF 3B

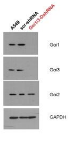

SF 3D

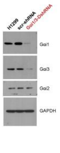

SF 4B

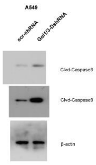

Figure 7A

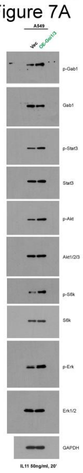

Figure 7B

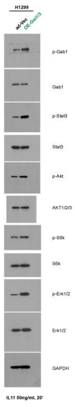

Figure 8F

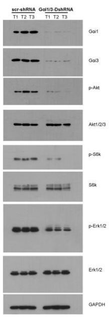

Figure 9B

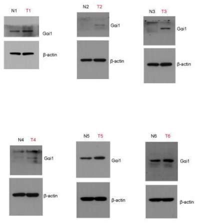

SF 3F

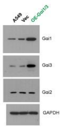

SF 3H

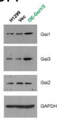

Supplement: Supplementary file 3 — The uncropped blotting images of the study. [file 41419_2026_8637_MOESM3_ESM.pdf]
